# Chronic nitric oxide mediates dual-layer gene regulation through mRNA m^6^A positional remodeling and parallel transcriptional reprogramming

**DOI:** 10.1016/j.redox.2026.104344

**Published:** 2026-08-16

**Authors:** Sara Shayan, Hannah Petraitis Kuschman, Christopher G. Kevil, Douglas D. Thomas

**Affiliations:** a University of Illinois Chicago, College of Pharmacy, Department of Pharmaceutical Sciences, Chicago, IL, USA; b LSU Health Shreveport, Departments of Pathology and Translational Pathobiology, Molecular & Cellular Physiology, and Cell Biology & Anatomy, Shreveport, LA, USA

**Keywords:** *N*^*6*^-methyladenosine, Nitric oxide, Epitranscriptome, mRNA, FTO, Triple-negative breast cancer

## Abstract

Nitric oxide (NO) is a pleiotropic free radical that functions as a master regulator of gene expression, and its sustained production within the tumor microenvironment reshapes the epitranscriptomic state of cancer cells. We previously demonstrated that NO inhibits the m^6^A mRNA demethylases FTO and ALKBH5 through dinitrosyliron complex formation while leaving the methyltransferase METTL3 intact, a demethylase-specific perturbation that increases global m^6^A on mRNA. Here, integrating m^6^A-RIP-seq and RNA-seq from triple-negative breast cancer cells, we show that chronic NO does not produce the uniform hypermethylation anticipated from demethylase inhibition. Instead, it redistributes m^6^A on mRNA, enriching the 5′UTR and coding sequence while depleting the 3′UTR and departing from the canonical stop-codon and 3′UTR topology. We found that the position of m^6^A, rather than its intensity or mere presence, shapes the outcome, in part by determining which reader protein is predicted to recognize it. In parallel, NO drives a canonical NF-κB and inflammatory transcriptional program. The transcriptional program is independent of the m^6^A methylome in both which genes respond and how strongly they respond, ruling out a linear methylome-to-transcriptome cascade; even so, m^6^A position remains associated with the direction of change among responding transcripts. The 3′UTR is the primary site of m^6^A loss and shows a suggestive computational link to miRNA-mediated regulation. Sense-antisense coordination reinforces the transcriptional response without bridging the two programs. These findings demonstrate that NO not only increases m^6^A abundance, but it also rewrites the m^6^A positional code, establishing spatial reprogramming of the epitranscriptome as a previously unrecognized mode of gene regulation.

## Highlights

• Chronic NO redistributes mRNA m^6^A instead of uniformly raising its abundance.
• m^6^A shifts into the 5′UTR and CDS while the 3′UTR loses the mark.
• Mark position and architecture, not abundance, dictate the functional outcome.
• NO m^6^A topology departs from the canonical stop-codon and 3′UTR pattern.
• An NF-κB transcriptional program runs parallel to, but decoupled from, the m^6^A code.

## Graphical abstract

**Figure:**
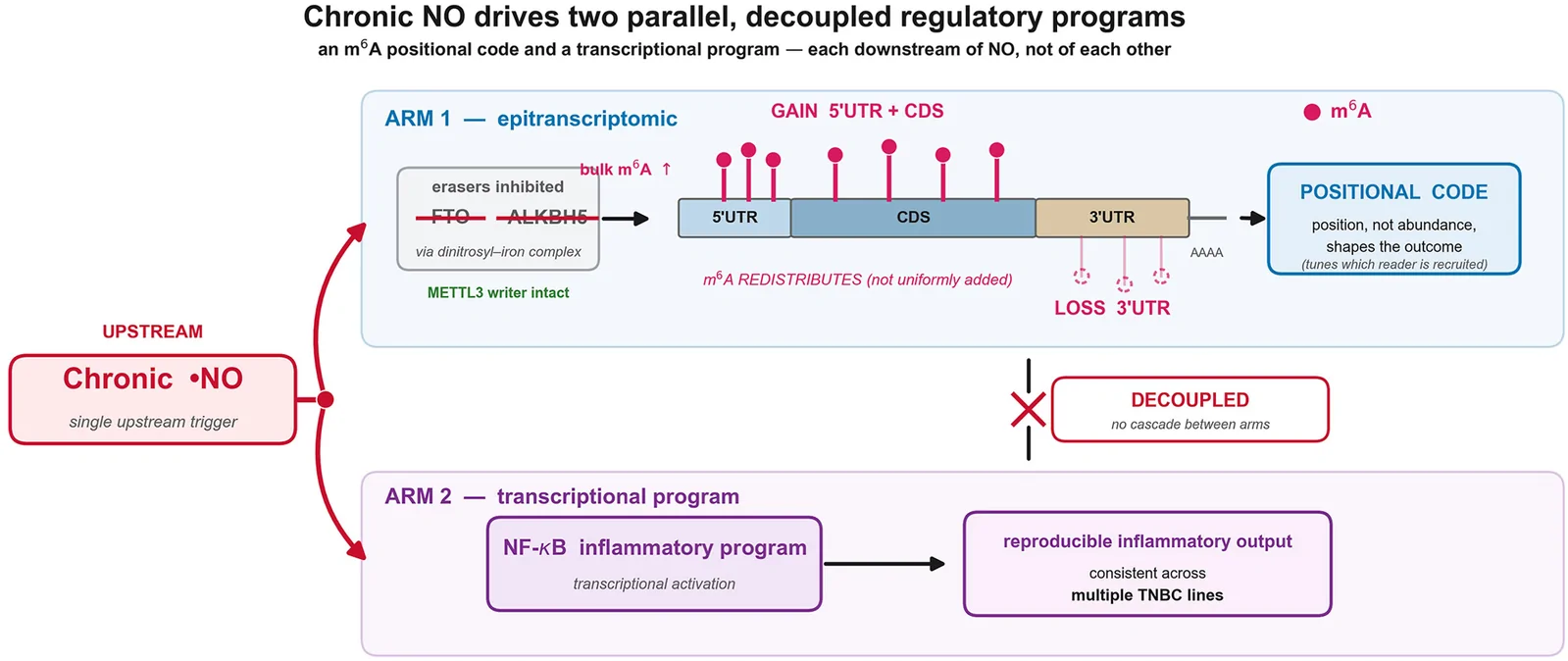

## 1 Introduction

Nitric oxide (NO) is an essential biological free radical that functions as a rapid signaling molecule by either directly binding to metal centers, such as the activation of soluble guanylate cyclase (sGC), or by forming nitrogen oxide-based covalent protein modifications (i.e. *S*-nitrosothiols (RSNO)) to mediate fundamental physiological processes including vasodilation, neurotransmission, and host defense [[1], [2], [3], [4], [5], [6], [7]]. Beyond classical signaling mechanisms our laboratory has established NO as an endogenous regulator of epigenetic methylation through direct inhibition of the Fe(II)/2-oxoglutarate-dependent dioxygenase (2-ODD) family of demethylases. We demonstrated that physiological NO concentrations directly and reversibly inhibit 2-ODD enzymes responsible for removing methyl groups from histones (e.g., KDM3A) [8,9], mRNA (e.g., FTO, ALKBH5) [10], and DNA (e.g., TET enzymes, ALKBH2) [11], leading to substrate-specific hypermethylation and altered gene expression.

In addition to NO's important biological functions dysregulated NO production (increased amount or duration of exposure) is associated with numerous pathological conditions, yet canonical NO signaling mechanisms cannot explain the vast number of transcriptional changes associated with NO-driven disease states [7,12]. Chronic NO exposure, however, sustained over days to weeks engages a fundamentally distinct biology, driving stable cellular reprogramming that extends beyond transient signaling to encompass durable epigenetic and epitranscriptomic modifications [10,13]. This distinction carries particular weight in many disease states including cancers where increased NO production, due to the upregulation of the inducible form of nitric oxide synthase (iNOS/NOS2), is associated with tumor-permissive gene expression, poor patient outcome, increased mortality, and resistance to chemotherapy across diverse cancer types [3,14,15]; including triple-negative breast (TNBC) [[16], [17], [18], [19], [20], [21], [22], [23], [24], [25], [26], [27], [28]], lung [[29], [30], [31]], prostate [[32], [33], [34]], brain [35,36], colon [[37], [38], [39]], melanoma [[40], [41], [42], [43]], and liver cancers [44,45]. Because the tumor microenvironment sustains NO flux over prolonged periods, chronic-exposure models are essential for capturing the full spectrum of NO-driven gene regulatory change.

*N* [6]-methyladenosine (m^6^A) is the most abundant internal modification of mammalian mRNA, present at an estimated 3–5 marks per polyadenylated transcript [46,47]. Transcriptome-wide mapping established early that m^6^A is not distributed uniformly along mRNA but is predominantly concentrated near the stop codon and within the 3′UTR [48]. The present data are consistent with this canonical topology, since the majority of m^6^A peaks reside in the 3′UTR (∼12,500 peaks) rather than the 5′UTR (∼1870 peaks); against this stable baseline, the redistribution we describe below represents a departure from the canonical distribution rather than a uniform amplification of it. m^6^A is installed by the METTL3/METTL14 methyltransferase complex [49], and removed by two 2-ODD mRNA demethylases: FTO (fat mass and obesity-associated protein) and ALKBH5 [50,51]. The functional consequences of m^6^A on mRNA are interpreted by reader proteins of distinct subcellular localization; nuclear YTHDC1 governs mRNA splicing and export [52], cytoplasmic YTHDF1 and YTHDF2 promote translation and decay, respectively [53], and the IGF2BP family stabilizes m^6^A-modified transcripts [54], with recent work demonstrating that the YTHDF paralogs act redundantly in mediating m^6^A-dependent degradation [55]. Critically, these outcomes are position-dependent: marks in the 5′UTR facilitate cap-independent translation, those within the coding DNA sequence (CDS) modulate translation elongation, and 3′UTR modifications predominantly influence mRNA decay and miRNA-mediated silencing [56].

We recently demonstrated that NO directly inhibits FTO demethylase activity by forming a dinitrosyliron complex (DNIC) at the enzyme's catalytic non-heme iron center, producing global m^6^A hypermethylation across six cancer cell lines [10]. This DNIC-mediated mechanism is shared by both mRNA demethylases, since FTO and ALKBH5 depend on the same Fe(II) cofactor for catalysis [57]. We previously demonstrated that the protein levels of FTO and ALKBH5 are unchanged by chronic NO. This indicates that the effect is demethylase-specific rather than restricted to FTO alone, and that it reflects catalytic inhibition rather than altered enzyme abundance. METTL3, the catalytic subunit of the methyltransferase writer complex, was by contrast unaffected by NO in both expression and activity, consistent with a selective perturbation of the eraser arm. Accordingly, methyltransferase inhibition did not prevent the NO-mediated increase in m^6^A-mRNA. In TNBC cells chronically treated with NO for 10 days, m^6^A-RIP-seq revealed widespread remodeling of the epitranscriptomic landscape, with subsets of hyper- and hypomethylated transcripts showing corresponding changes in gene expression [10].

Despite establishing the definitive mechanism for NO-mediated increases in bulk m^6^A content, that original analysis treated the mRNA modification as essentially binary, present or absent, and left unaddressed how the spatial organization of marks across transcript architecture might itself encode regulatory information. Several critical questions therefore remained open. Does peak location within the transcript predict expression outcome? Do multiple peaks on the same transcript act additively, synergistically, or redundantly? Can the pattern of m^6^A marks predict which reader protein is sequence-compatible? And does the epitranscriptomic spatial code propagate into the steady-state transcriptome, or does it operate as a regulatory layer parallel to, and decoupled from, transcriptional control? To answer these questions, we return to the datasets from *Kuschman* et al. [10] to perform entirely new analyses not included in the original publication. Specifically, we performed a comprehensive multi-omics analysis integrating m^6^A-RIP-seq and RNA-seq from the chronic NO exposure model. By mapping 49,111 m^6^A peaks across 9612 genes onto detailed transcript architecture, including a novel CDS subdivision analysis, we developed a positional code framework linking m^6^A spatial patterns to expression outcomes. Integrating sequence-based reader assignment, pathway and transcription factor enrichment, and cross-cell-line analysis, we reveal that chronic NO rewires the epitranscriptomic landscape not as a simple change in abundance, but as a spatial regulatory code with distinct functional consequences at each transcript position.

## 2 Materials and Methods

### 2.1 Datasets and study design

All data in this study are derived from the experiments described in our original study Kushman *et, al.* m^6^A-RIP-seq, RNA-seq, and associated data were from MDA-MB-231 cells treated with DETA/NO (100 μM, 10 days) [10]. For the chronic NO exposure, DETA/NO was replenished rather than added once: cells were plated at 1.5 × 10^6^ per 100 mm dish, treated with 100 μM DETA/NO after adherence, and every 48 h were trypsinized, pooled by replicate, counted, and re-plated at the same density with fresh 100 μM DETA/NO, for approximately five dosing cycles across the 10-day course. This 48 h replenishment schedule was selected to sustain a low, physiologically relevant steady-state NO concentration on the order of 10–500 nM in cells given the finite half-life of the donor, rather than to deliver a supraphysiologic bolus; this range lies at the low end of the interval over which NO inhibits the Fe(II)/2-oxoglutarate-dependent mRNA demethylases FTO and ALKBH5 in our prior enzymatic and cellular studies [10]. Raw data are deposited under GEO SuperSeries GSE236272: GSE236270 (MDA-MB-231 RNA-seq, 3 replicates per condition), GSE236271 (MDA-MB-231 MeRIP-seq), and GSE236269 (MDA-MB-468 RNA-seq, 3 replicates per condition). Alignment to GRCh38 (Ensembl v109) and peak calling using the exomePeak algorithm [58] were performed yielding 49,111 peaks across 9612 genes (Table 1).

**Table 1:** Summary of multi-omics datasets analyzed. m^6^A-RIP-seq, RNA-seq, and proteomics data from MDA-MB-231 and MDA-MB-468 cells chronically treated with DETA/NO (100 μM, 10 days).

Data Type	Cell Line	Treatment	Replicates	Peaks/Genes	Platform
m^6^A-RIP-seq	MDA-MB-231	DETA/NO 100uM, 10d	2	49,111 peaks/9612 genes	Illumina HiSeq
RNA-seq	MDA-MB-231	DETA/NO 100uM, 10d	3	36,472 genes	Illumina HiSeq
RNA-seq	MDA-MB-468	DETA/NO 100uM, 10d	3	36,472 genes	Illumina HiSeq
Proteomics	MDA-MB-231	DETA/NO 100uM, 10d	3	2158 proteins	Spectral counting
Proteomics	MDA-MB-468	DETA/NO 100uM, 10d	3	2158 proteins	Spectral counting

### 2.2 Peak annotation and gene-level summarization

m^6^A peaks were annotated to nine transcript region categories (5′UTR, start codon, CDS first 25%, CDS middle 50%, CDS last 25%, stop codon, 3′UTR, intronic, intergenic) based on midpoint overlap with Ensembl v109 canonical transcript features. CDS subdivision used fractional positioning within the coding sequence. Per-gene metrics included regional peak counts, fractions, mean log^2^ enrichment ratio, binary location signatures, and the Herfindahl–Hirschman concentration index (HHI).

### 2.3 Differential expression

RNA-seq differential expression was computed using pyDESeq2 v0.5.4 [59], a Python implementation of the DESeq2 statistical framework [60], with Wald test and Benjamini–Hochberg FDR correction. Differential m^6^A methylation was assessed under the n = 2 per-condition design; of 49,111 annotated peaks, 64 reached adjusted significance (FDR < 0.05). Spatial-distribution analyses therefore aggregate across the full threshold-defined peak population (∼20,400 peaks at |log2RR| > 0.2) rather than relying on individually significant peaks.

The effect-size threshold for significant m^6^A change (|mean log2RR| > 0.2) was confirmed by sensitivity analysis across the interval 0.10–0.30. The three spatial-polarization enrichments—5′UTR hypermethylation, CDS hypermethylation, and 3′UTR hypomethylation, retained their direction and statistical significance at every threshold tested (Fisher exact test, exonic-mRNA background; all *P* < 1 × 10^−12^; no 95% confidence interval crossed unity). Odds-ratio point estimates were stable to within rounding across the interval (5′UTR 2.49–2.58, CDS 1.29–1.36, 3′UTR 1.97–2.15), with the locked-threshold values of 2.56, 1.36, and 2.07, respectively. The m^6^A-responsive, m^6^A-independent, and m^6^A-only gene partitions were likewise recovered at all thresholds. Because the conclusions are insensitive to the precise cutoff within this range, the 0.2 threshold represents a representative interior value rather than a point selected to maximize the observed effect, supporting the robustness of the spatial-code model (Fig. S2).

### 2.4 Predictive modeling

Eight model variants were tested: null, baseMean-only, m^6^A-only (Linear/RF/XGBoost), and full (Linear/RF/XGBoost). Five-fold stratified cross-validation was performed on log^2^FoldChange quintile bins with 24 m^6^A features and 1 expression covariate. Significance was assessed by permutation test (1000 shuffles of target variable). Feature importance was evaluated using SHAP (v0.50.0).

### 2.5 Reader assignment

Sequence-based reader compatibility scores were computed per peak using published binding motifs: YTHDC1 (UGACA) [52], YTHDF2 (GGACU) [61], YTHDF1 (GGACU + structural context) [62], and IGF2BP (CAUAC) [54]. Scores are binary (0/1) and represent sequence compatibility, not condition-specific binding.

### 2.6 Pathway and transcription factor enrichment

Over-representation analysis (ORA) used GSEApy v1.1.11^63^ with GO Biological Process 2023, KEGG 2019, MSigDB Hallmark 2020, and Reactome 2022 libraries. Pre-ranked GSEA used log^2^FoldChange as the ranking metric with 1000 permutations [63]. For cross-line concordance, pre-ranked GSEA was run on the MDA-MB-231 and MDA-MB-468 differential-expression rankings separately (ranking metric sign(log2FC) × −log10 *P*; minimum 15/maximum 500 genes; 1000 permutations; seed 42), and per-pathway normalized enrichment scores were compared by Spearman correlation over shared pathways. Significance of the cross-line concordance was assessed by a gene-label permutation null (1000 shuffles). To make the permutation null computationally tractable, the null distribution was generated with a validated analytic weighted Kolmogorov–Smirnov enrichment-score surrogate (validated against exact GSEApy NES on the observed ranking at Spearman ρ = 0.93/Pearson *r* = 0.97); the reported observed concordance uses exact GSEApy NES. TF enrichment used ENCODE/ChEA Consensus TFs, TRRUST 2019, TF Perturbations, and ENCODE TF ChIP-seq 2015 libraries. FDR correction (Benjamini–Hochberg, adjusted *P* < 0.05).

### 2.7 Co-methylation module clustering

Genes were partitioned by their m^6^A spatial architecture using a ten-feature per-gene vector: peak counts in the 5′UTR, CDS, 3′UTR, and intronic regions; peak fractions in the 5′UTR, CDS, and 3′UTR; the mean per-peak log2 risk-ratio, capturing methylation magnitude and direction; the Herfindahl–Hirschman concentration index (HHI) of peak distribution across regions; and the number of distinct regions occupied. Features were standardized to zero mean and unit variance, and genes were clustered by K-means (n_init = 20, fixed random seed). The number of clusters was selected by silhouette score over k = 3–8, which returned k = 8; silhouette scores were modest throughout this range (≈0.24), so the resulting modules should be interpreted as descriptive partitions of m^6^A spatial architecture rather than sharply separated clusters. Expression (log2 fold-change) was excluded from the feature set and tested separately as an outcome, so that any association between architecture and expression was not built into the partition. Principal-component projection of the standardized features was used for visualization only and did not inform cluster assignment.

### 2.8 Multi-omics integration

A unified table (9612 genes × 76 columns) merged m^6^A, RNA-seq, CDS subdivision, and reader profiles. Bivariate and partial Spearman correlations with bootstrap 95% CI (1000 resamples) assessed cross-layer relationships.

### 2.9 Sequence and motif analysis

Peak sequences (101 bp centered on midpoint) were extracted from GRCh38 using pyfaidx v0.9.0.3. DRACH motifs (D = [AGU], R = [AG], A, C, H = [AUC]) were scanned by regex. GC content was computed per window. K-mer enrichment (k = 5) used Fisher's exact test with BH-FDR correction.

### 2.10 Peak clustering and co-methylation modules

Peaks within the same gene were sorted by midpoint, and inter-peak distances were computed. Peaks with nearest-neighbor distance ≤500 bp were classified as clustered. Co-methylation modules were identified by K-means clustering (k = 3–8, optimal by silhouette score) of a 10-feature gene matrix (regional peak counts, fractions, mean log^2^RR, HHI, regions occupied) after StandardScaler normalization.

### 2.11 BigWig signal visualization

RPKM-normalized BigWig coverage files (20 bp bin resolution) were generated from aligned BAM files for all four MeRIP-seq libraries (2 replicates × 2 conditions) for both IP and input fractions. Signal extraction was performed using pyBigWig (v0.3.22). For metagene profiles, IP and input signal were extracted in a ±50-bin window centered on each peak midpoint, log^2^(IP/Input + 0.1) enrichment ratios were computed, and profiles were averaged across all peaks within each transcript region. Gaussian smoothing (σ = 3 bins) was applied for visualization. For gene-level browser tracks, signal was extracted at single-nucleotide resolution across annotated exons, with introns collapsed to produce linearized transcript views. Signal from replicate BigWig files was averaged per condition. Peak topology analysis used ±10 kb windows with 400-bin resolution, sampling up to 800 peaks per region. Peak isolation was defined using a 2 kb nearest-neighbor distance threshold on same-gene peaks. Local peak density was computed as the number of same-gene peaks within ±5 kb. Signal heatmaps display ±50-bin windows for up to 4003 peaks (sampled proportionally by region), sorted by transcript region and within-region signal intensity.

### 2.12 miRNA target enrichment

Gene sets defined by 3′UTR peak direction (hypo: log^2^RR < −0.2; hyper: log^2^RR > 0.2) were tested for miRNA target enrichment using GSEApy enrich() [64] with TargetScan microRNA 2017 and miRTarBase 2017 libraries against a background of 9612 gene symbols.

### 2.13 Sense–antisense analysis and convergence testing

Sense–antisense gene pairs were identified by parsing the Ensembl v109 GTF annotation for opposite-strand gene pairs with overlapping genomic coordinates. A total of 2697 pairs were classified by m^6^A status as both-m^6^A (987), asymmetric (1,270), or neither (440). For the convergence test, genes in asymmetric pairs were classified into the two-programs categories (m^6^A-responsive, m^6^A-independent, m^6^A-only, Neither) using thresholds of |mean log^2^RR| > 0.2 and adjusted *P* < 0.05. Bridge pair enrichment was assessed by binomial test against the expected proportion under independence. Expression coordination was measured by Spearman correlation between m^6^A gene and partner gene log^2^FC, stratified by category, with bootstrap 95% CIs (1000 resamples). Concordance rates were compared across categories by χ^2^ test, and correlation differences were assessed by Fisher z-transformation. Sensitivity analysis varied thresholds across 15 combinations (|log^2^RR| ∈ {0.1, 0.15, 0.2, 0.25, 0.3}; adjusted *P* ∈ {0.01, 0.05, 0.10}).

### 2.14 Statistical analysis

Statistical tests included Fisher's exact test (2 × 2 contingency), χ^2^ test (r × c contingency), Mann–Whitney U (two-group comparisons), Kruskal–Wallis (multi-group), Wilcoxon signed-rank (one-sample), Spearman rank correlation, partial Spearman correlation, Kolmogorov–Smirnov test, Fisher z-test for correlation comparison, *F*-test for segmented regression, and permutation testing (1000 iterations). Multiple testing correction used Benjamini–Hochberg FDR where noted. All analyses were performed in Python 3.13.5 using scipy, scikit-learn (v1.8.0), statsmodels, XGBoost (v3.2.0), and custom scripts.

## 3 Results

### 3.1 Chronic NO remodels the m^6^A mRNA architecture

In this study we integrated two genome-wide datasets generated from a chronic NO-exposure model: triple-negative breast cancer cells MDA-MB-231 (with MDA-MB-468 RNA-seq for cross-line validation) were treated with the slow-release NO donor DETA/NO (100 μM) to generate prolonged steady-state physiologic concentrations of NO for 10 days. At 10 days, the cells were harvested and profiled for transcriptome-wide m^6^A distribution and steady-state gene expression (m^6^A-RIP-seq and matched from RNA-seq; see Materials and Methods for the NO-treatment and sequencing details [10]). Rather than the uniform hypermethylation anticipated from mRNA demethylase inhibition, chronic NO redistributed this elevated landscape into directional preferences along the transcript, producing a net gain at the 5′ end and a loss at the 3′ end. Hypermethylated peaks were significantly enriched at the 5′UTR (OR = 2.56, 95% CI [2.32–2.82], *P* = 1 × 10^−71^) and the coding sequence (CDS) (OR = 1.36, 95% CI [1.29–1.44], *P* = 1 × 10^−25^), whereas hypomethylated peaks predominated at the 3′UTR (OR = 2.07, 95% CI [1.96–2.18], *P* = 3 × 10^−147^; Fig. 1A; Table 2). The Kolmogorov–Smirnov test confirmed that hyper- and hypomethylated peaks occupy fundamentally different regional distributions (*D* = 0.419, *P* = 5.0 × 10^−78^). The spatial-distribution analyzed across the full peak population rather than relying on individually significant peaks (see Table 3).

**Fig. 1:**
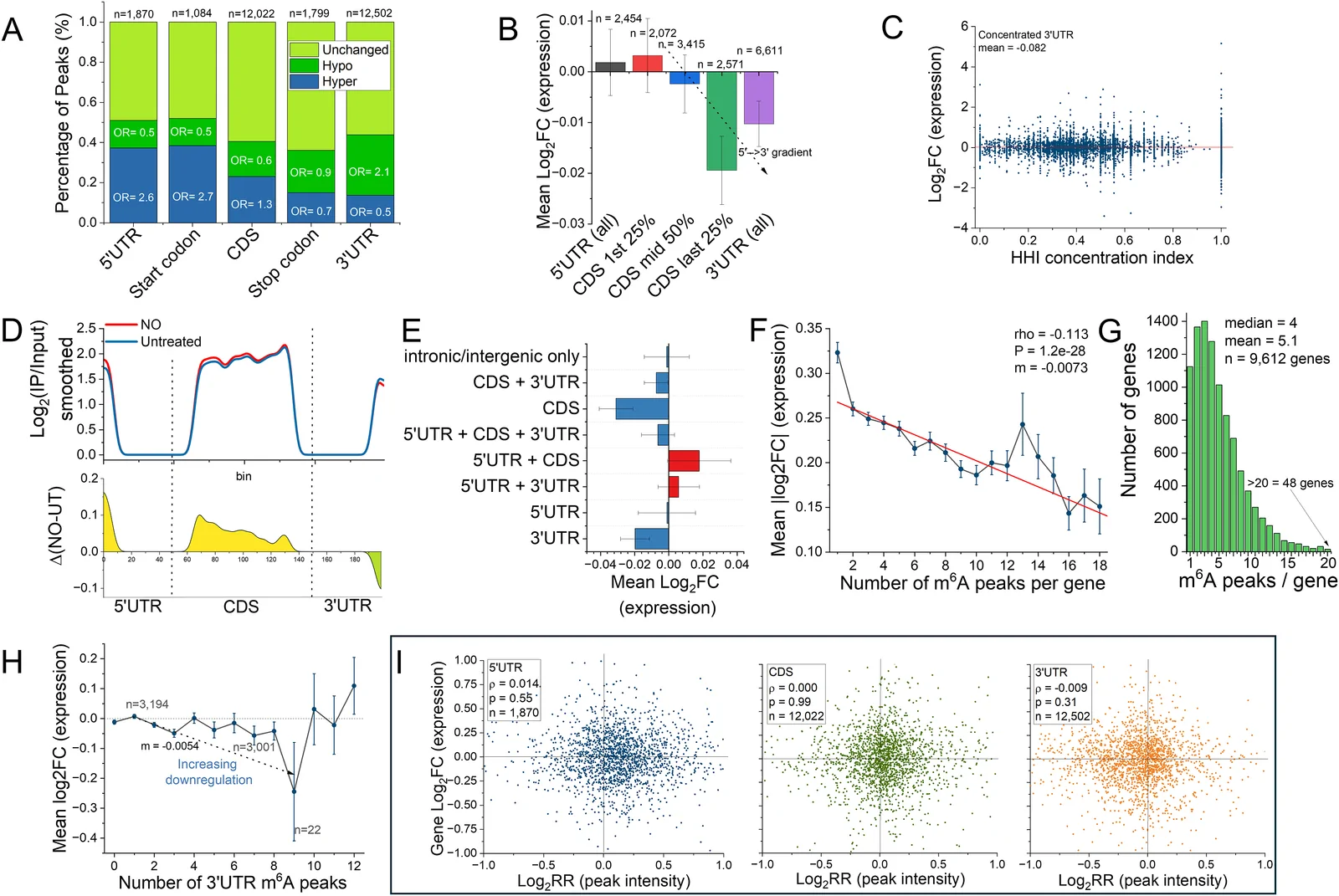
**Chronic NO remodels m^6^A architecture, not just abundance.** (**A**) Regional distribution of m^6^A peaks by direction (hyper, hypo, unchanged). Hypermethylation is enriched at the 5′UTR and CDS while hypomethylation predominates at the 3′UTR. (**B)** CDS subdivision into positional quartiles, showing the mean expression log2 fold-change (±SEM) for genes with m^6^A peaks in each region (5′UTR, CDS first 25%, CDS middle 50%, CDS last 25%, 3′UTR). Peaks in the proximal CDS associate with modest upregulation and peaks in the distal CDS and 3′UTR with downregulation, defining a significant 5′→3′ gradient (Jonckheere–Terpstra p = 0.004). (**C**) Per-gene expression log2 fold-change versus the Herfindahl–Hirschman concentration index of m^6^A peak distribution (blue points, all genes; red line, binned mean). Genes with marks concentrated in the 3′UTR show amplified downregulation (mean log2FC = −0.082) relative to genes with distributed marks (−0.001; Mann–Whitney P = 1.1 × 10−4). (**D**) Metagene m^6^A enrichment profiles by transcript region. (Upper) Average log^2^(IP/Input) enrichment signal from RPKM-normalized MeRIP-seq BigWig coverage across all annotated m^6^A peaks, plotted as a metagene profile spanning the 5′UTR, CDS, and 3′UTR (separated by dashed vertical lines). Red line: NO-treated; Blue line: untreated. (Lower) Differential enrichment (Δ = NO – UT). Positive values (yellow shading) indicate hypermethylation; negative values (green shading) indicate hypomethylation. Gaussian smoothing (σ = 3) applied. (**E**) Location signature showing mean expression change and percentage of significantly up/down-regulated genes for each combinatorial signature. (**F**) Multiplicity dose-response: mean absolute fold change decreases with increasing peak count. (**G**) Frequency distribution of m^6^A peaks per gene across the 9612 detected genes; the distribution is right-skewed (median 4 peaks, range 1 to 53), with roughly 40% of genes carrying three or fewer peaks. (**H**) 3′UTR-specific dose-response showing progressive downregulation with increasing 3′UTR peak count. (**I**) Individual peak intensity (log^2^ enrichment ratio) does not correlate with host gene expression change in any transcript region.

**Table 2:** Regional distribution of m^6^A peaks (peak landscape). Peak counts and percentages by transcript region for hyper-, hypo-, and unchanged-methylation directions across the full 49,111-peak population.

Direction	Region	N peaks	% of direction	% of total
hyper	5UTR	1115	11.1	2.3
hyper	CDS	2767	27.5	5.6
hyper	3UTR	1992	19.8	4.1
hyper	intronic	3486	34.7	7.1
hyper	intergenic	668	6.6	1.4
hypo	5UTR	404	3.9	0.8
hypo	CDS	2108	20.3	4.3
hypo	3UTR	4142	40.0	8.4
hypo	intronic	2988	28.8	6.1
hypo	intergenic	707	6.8	1.4
unchanged	5UTR	1435	5.0	2.9
unchanged	CDS	7147	24.9	14.6
unchanged	3UTR	8167	28.5	16.6
unchanged	intronic	10317	35.9	21.0
unchanged	intergenic	1585	5.5	3.2

**Table 3:** Key statistical results across the spatial-code analysis. Test, statistic, P-value, sample size, and supporting main figure for each principal claim.

Claim	Test	Statistic	P-value	n	Figure
Hyper enriched at 5UTR	Fisher's exact	OR = 2.56	1e-71	49111	Fig. 1
Hypo enriched at 3UTR	Fisher's exact	OR = 2.07	3e-147	49111	Fig. 1
Hyper vs hypo distribution different	KS test	D = 0.419	5.0e-78	49111	Fig. 1
CDS gradient exists	Spearman trend	rho = −0.019	0.013	9612	Fig. 1
Concentration amplifies expression	Partial correlation	coef = 0.067	1.5e-9	9612	Fig. 1
3UTR concentration drives downreg	Mann-Whitney	FC = −0.082 vs −0.001	1.1e-4	850	Fig. 1
Location signatures predict expression	Chi-square + permutation	chi2 = 28.4	0.012	9612	Fig. 1
5UTR-containing - > upregulation	Fisher's exact	OR = 1.23	3.7e-4	9612	Fig. 1
Multiplicity buffers expression	Spearman	rho = −0.113	1.2e-28	9612	Fig. 1
3UTR dose-dependent downregulation	Spearman + MW	rho = −0.037	0.0008 (MW)	9612	Fig. 1
Predictive model significant	Permutation (1000x)	rho = 0.058	0.001	9612	Fig. 1
Individual peak intensity NOT predictive	Spearman (6 regions)	all rhõ0	all >0.12	49111	Fig. 1
Concordant peaks still buffer	Spearman	rho = −0.080	1.5e-13	8488	Fig. 1
42.6% peaks spatially clustered	Descriptive	20,924/49,111	N/A	49111	Fig. 3
Clustered peaks stronger m^6^A	Mann-Whitney	|RR| 0.184 vs 0.138	2.9e-125	49111	Fig. 3
YTHDF2 enriched in clusters	Fisher's exact	OR = 2.56	∼0	49111	Fig. 3
YTHDC1 depleted from clusters	Fisher's exact	OR = 0.32	∼0	49111	Fig. 3
YTHDC1-expression positive	Spearman	rho = +0.036	5.7e-4	9612	Fig. 3
YTHDF2-expression negative	Spearman	rho = −0.056	6.6e-6	9612	Fig. 3
NO shifts YTHDC1 up	Wilcoxon	delta = +0.031	3.6e-27	9612	Fig. 3
NO shifts YTHDF2 down	Wilcoxon	delta = −0.024	7.1e-91	9612	Fig. 3
YTHDC1-favored in Discordant-Methyl	Fisher's exact	OR = 2.87	<0.0001	9612	Fig. 3
m^6^A-responsive larger RNA changes	Mann-Whitney	|FC| 0.334 vs 0.302	4.8e-7	3978	Fig. 4
m^6^A-independent: 117 pathways	gseapy ORA	117 terms FDR<0.05	various	3080	Fig. 4
m^6^A-responsive: only 6 pathways	gseapy ORA	6 terms FDR<0.05	various	898	Fig. 4
m^6^A-independent: 1425 TFs	gseapy ORA (4 TF databases)	1425 sig TFs	various	3080	Fig. 4
Cross-line program replication	Spearman	rho = 0.39/0.37/0.19	<2e-12	9612	Fig. 4
Cross-line GSEA concordance	Spearman (permutation)	rho = 0.318	0.001	3115	Fig. 4
3UTR hypo more miRNA targets	Fisher's exact	OR = 1.83	<0.0001	4606	Fig. 5
Module 4 strongest downregulation	Wilcoxon signed-rank	median FC = −0.047	1e-4	906	Fig. 5
8 modules differ in expression	Kruskal-Wallis	H = 20.7	4.2e-3	9612	Fig. 5
Module x concordance associated	Chi-square	chi2 = 296.1	6.0e-40	9612	Fig. 5

Subdivision of the CDS into positional quartiles revealed a gradient in the m^6^A effect on expression: peaks within the first 25% of the CDS were associated with modest upregulation (mean log2FC = +0.003), while peaks within the last 25% were associated with downregulation (mean log2FC = −0.019), mirroring the behavior of 3′UTR peaks (Fig. 1B–D). This 5′→3′ gradient was a statistically significant monotonic trend by the Jonckheere–Terpstra test for ordered position (p = 0.004; Spearman ρ = −0.019, P = 0.013). Although of small effect size, accounting for less than 0.1% of per-gene variance in expression change, it is consistent with a functional transition within the CDS in which the distal coding region may behave analogously to the 3′UTR.

The spatial concentration of m^6^A marks within individual genes further modulated expression outcome. To determine whether spreading marks across multiple transcript regions, versus concentrating them in one, produces different outcomes, we quantified regional concentration using the Herfindahl–Hirschman Index (HHI), which ranges from 0 (marks distributed evenly across regions) to 1 (all marks within a single region). Concentrated m^6^A, particularly at the 3′UTR, significantly amplified downregulation. Genes with concentrated 3′UTR modifications showed a mean log2FC of −0.082, compared with −0.001 for genes with distributed modifications (Mann–Whitney *P* = 1.1 × 10^−4^; Fig. 1C). Partial correlation confirmed that concentration independently predicted effect size after controlling for peak count (P = 1.5 × 10^−9^), with the effect most pronounced among genes whose marks concentrate in the 3′UTR. The surrounding sequence context tracked the spatial pattern: hypermethylated regions were GC-rich (55.9%) while hypomethylated regions were AU-rich (47.0%), with the region-resolved GC content shown in Supplementary Fig. S1A. This same compositional split was reflected at the motif level, where hypermethylated peaks favored GC-containing DRACH variants such as GGACC and GC-rich k-mers whereas hypomethylated peaks favored AU-rich DRACH variants and k-mers, with DRACH-motif prevalence resolved by transcript region and methylation direction shown alongside these enrichments (Supplementary Figs. S1B, C, D). Because GC content is itself an inherent property of 5′ versus 3′ transcript regions, this correlation may reflect baseline regional composition rather than NO-specific remodeling; it nonetheless suggests that local sequence composition, which governs RNA secondary structure and protein binding accessibility, could help organize the directional m^6^A landscape. How demethylase inhibition produces a localized loss of m^6^A at the 3′UTR rather than a uniform gain remains to be established; we return to a possible explanation in the Discussion.

To validate the spatial polarization of m^6^A against the underlying sequencing signal, we computed metagene enrichment profiles from RPKM-normalized BigWig coverage across all annotated peaks. For each peak, we extracted IP and input signal in a ±50-bin window centered on the peak midpoint, computed the log2(IP/Input) enrichment ratio, and averaged across all peaks within each transcript region (5′UTR, CDS, 3′UTR), separately for NO-treated and untreated conditions (Fig. 1D, upper panel). These profiles confirmed that m^6^A enrichment is strongest at the 5′UTR (peak log2 ratio = 1.88 in NO versus 1.72 in UT) and the CDS (2.17 versus 2.13), with markedly lower enrichment at the 3′UTR (1.43 versus 1.50). Critically, the NO–UT difference (Δ enrichment, lower panel) was positive across the 5′UTR (Δ = +0.16) and the 5′ portion of the CDS (Δ = +0.10), consistent with hypermethylation at these positions, yet turned negative across the 3′UTR (Δ = −0.03), consistent with net hypomethylation. This direct visualization recapitulates the spatial polarization identified by differential peak analysis and provides independent confirmation, from continuous coverage data, that chronic NO drives m^6^A gain at the transcript 5′ end and loss at the 3′ end.

### 3.2 m^6^A location and multiplicity define a spatial code

We next asked whether m^6^A spatial patterns could systematically predict expression outcome. Binary location signatures, encoding which transcript regions harbored peaks for each gene, significantly predicted the direction of expression change (χ^2^ = 28.4, *P* = 0.012). Genes with 5′UTR + CDS signatures showed the strongest upregulation (mean log2FC = +0.018, 23.4% significantly up), whereas CDS-only signatures were the most downregulated (mean log2FC = −0.031, 22.2% significantly down), followed by 3′UTR-only signatures (−0.020, 23.2% significantly down;Fig. 1E). A permutation-based predictive model incorporating 24 m^6^A architectural features confirmed that spatial patterns significantly predicted expression outcome (*P* = 0.001).

Unexpectedly, increasing m^6^A multiplicity showed a weak inverse association with the magnitude of expression change (Spearman ρ = −0.113, P = 1.2 × 10^−28^; Fig. 1F); genes carrying more m^6^A peaks tended toward smaller, not larger, fold-changes. This is a small, graded tendency across the gene population (|ρ| ≤ 0.11) rather than a per-gene rule. However, most genes carried few m^6^A peaks, with a right-skewed distribution spanning 1 to 53 peaks per gene and a median of 4 (Fig. 1G). It is also largely accounted for by gene expression level; highly expressed genes carry more called peaks and show inherently smaller fold-changes, and controlling for expression level (baseMean) reduces the correlation to ρ ≈ −0.04. The apparent multiplicity effect therefore substantially reflects expression level rather than a graded m^6^A dose. Within the 3′UTR specifically, increasing peak number was associated with a similarly weak downregulation (slope −0.0054 per peak; ρ = −0.037, P = 2.4 × 10^−3^), again a small tendency rather than a per-gene rule (Fig. 1H).

Critically, individual peak intensity (log2 enrichment ratio) did not predict host-gene expression change in any transcript region (all Spearman ρ ≈ 0, *P* > 0.12;Fig. 1I). This dissociation between modification intensity and functional output establishes a key principle: the m^6^A regulatory signal is encoded in the architecture of the modification, where concentration denotes how tightly a transcript's marks are clustered within it (the HHI index) and magnitude denotes the intensity of an individual peak's change; the message is that architecture, namely position, number, and concentration, shapes expression rather than the magnitude of individual marks.

### 3.3 Gene-level visualization confirms the spatial code at individual loci

To illustrate the spatial code at individual loci, we visualized exon-level m^6^A enrichment from RPKM-normalized BigWig files across nine representative cancer-associated genes, selected to demonstrate each regional modification pattern alongside concordant expression change (Fig. 2). For each gene, the linearized transcript view shows log2(IP/Input) signal tracks for NO-treated (red) and untreated (blue) conditions, with annotated m^6^A peaks marked below. Three genes exemplified 5′UTR hypermethylation with upregulation (Fig. 2A): FGFR1 (fibroblast growth factor receptor 1; Δm^6^A = +0.24, log2FC = +0.18), a receptor tyrosine kinase amplified in breast cancer whose 5′UTR gain is consistent with enhanced cap-independent translation; STK11 (serine/threonine kinase 11/LKB1; Δm^6^A = +0.32, log2FC = +0.33), a tumor suppressor and master regulator of AMPK signaling; and GNG11 (G protein subunit gamma 11; Δm^6^A = +0.22, log2FC = +0.95), a G protein signaling component whose strong upregulation accompanied modest 5′UTR hypermethylation. In the browser tracks, the NO condition shows visibly elevated enrichment at the 5′UTR exons relative to UT, confirming the hypermethylation detected by peak calling.

**Fig. 2:**
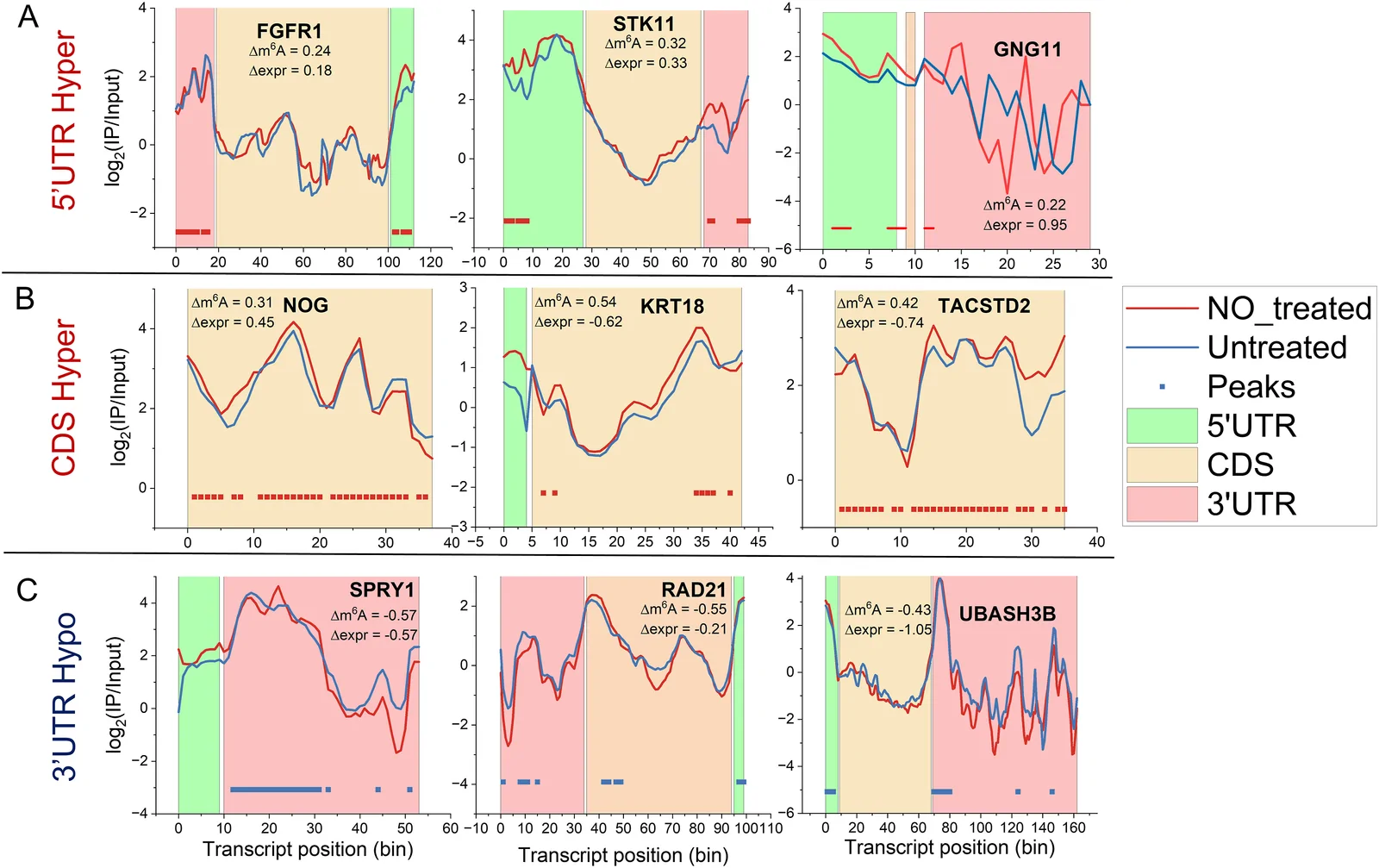
**Gene-level m^6^A browser tracks for representative cancer-associated genes.** Exon-only browser views of log^2^(IP/Input) MeRIP-seq signal from RPKM-normalized BigWig coverage at nine genes, organized by regional modification pattern. (**A**) 5′UTR hypermethylation with concordant upregulation: *FGFR1*, *STK11*, *GNG11*. (**B**) CDS hypermethylation: *NOG*, *KRT18*, *TACSTD2*. (**C**) 3′UTR hypomethylation with concordant downregulation: *SPRY1*, *RAD21*, *UBASH3B*. Red traces: NO-treated; blue traces: untreated. Colored squares below tracks indicate annotated m^6^A peak positions. Introns are collapsed to show exonic signal only. All genes were selected to satisfy three criteria: established cancer relevance, significant m^6^A change (|Δ enrichment| > 0.2), and concordant expression change in the expected biological direction.

Three CDS-hypermethylated genes illustrated the range of expression outcomes associated with coding-region modification (Fig. 2B): NOG (Noggin; Δm^6^A = +0.31, log2FC = +0.45), a BMP antagonist involved in EMT whose CDS hypermethylation accompanied upregulation rather than the downregulation seen in the other two, illustrating that CDS modifications do not uniformly suppress expression; KRT18 (keratin 18; Δm^6^A = +0.54, log2FC = −0.62), an epithelial differentiation marker whose downregulation supports EMT progression; and TACSTD2 (tumor-associated calcium signal transducer 2/Trop-2; Δm^6^A = +0.42, log2FC = −0.74), a clinically validated therapeutic target in TNBC. The CDS enrichment gain is clearly visible in the NO tracks spanning coding exons, though the differing outcomes across these three genes underscore that CDS position within the 5′→3′ gradient (Fig. 1B) modulates the functional consequence of CDS methylation.

Finally, three 3′UTR-hypomethylated genes showed concordant downregulation (Fig. 2C): SPRY1 (Sprouty RTK signaling antagonist 1; Δm^6^A = −0.57, log2FC = −0.57), whose loss of 3′UTR m^6^A is consistent with miRNA binding-site exposure and YTHDF2-mediated decay; RAD21 (cohesin complex component; Δm^6^A = −0.55, log2FC = −0.21), essential for genomic stability and sister-chromatid cohesion; and UBASH3B (ubiquitin-associated and SH3 domain-containing B; Δm^6^A = −0.43, log2FC = −1.05), a ubiquitin signaling regulator whose strong downregulation parallels the largest 3′UTR m^6^A loss. In each case, the UT browser tracks show higher 3′UTR enrichment than NO, visually confirming the loss of modification at these positions. Collectively, these gene-level visualizations demonstrate that the statistical patterns identified by our spatial code analysis, hypermethylation at the 5′UTR/CDS with upregulation and hypomethylation at the 3′UTR with downregulation, are directly visible in the raw MeRIP-seq signal at individual loci with established roles in cancer biology.

To provide transcript-level examples spanning the full positional code, we compiled a catalog of representative genes in which chronic NO produced a regional m^6^A change together with an expression change (Supplementary Table S7). These include transcripts that gain m^6^A within the first 25% of the CDS (for example SLC16A2, TENT5A, and the Trop-2 oncogene TACSTD2), transcripts that gain or retain m^6^A toward the last 25% of the CDS (for example the oncogene VEGFA and the breast-cancer coactivator NCOA3), and transcripts in which a 5′UTR or CDS m^6^A gain co-occurs with increased abundance (for example TNFAIP3/A20, NFKBIA, and ICAM1); cancer-relevant loci across this set include COSMIC Cancer Gene Census oncogenes and tumor suppressors such as KRAS, VEGFA, SMAD4, STK11, and DNMT3A. We present these as illustrative single-transcript instances of the positional code rather than as evidence of a causal methylation-to-expression rule, since SMAD4 gains 5′UTR m^6^A yet is downregulated, and the genome-wide analysis below shows that m^6^A change and expression change are decoupled at the gene level; the examples therefore show the positional code coinciding with altered expression at individual loci, not m^6^A directing the expression change.

### 3.4 Spatially clustered peaks are predicted to engage distinct reader machinery

To explore whether the spatial code connects to the reader machinery, we analyzed the spatial clustering of m^6^A peaks. Of these, 42.6% (20,924/49,111) of peaks were spatially clustered (≤500 bp from a neighboring peak), forming 6500 distinct clusters, and clustered peaks underwent larger NO-induced changes in methylation than isolated peaks (note that a peak is itself an m^6^A site, so the comparison is the magnitude of the NO-induced change at clustered versus isolated sites) (|log2RR| = 0.184 versus 0.138, Mann–Whitney *P* = 2.9 × 10^−125^).

**Fig. 3:**
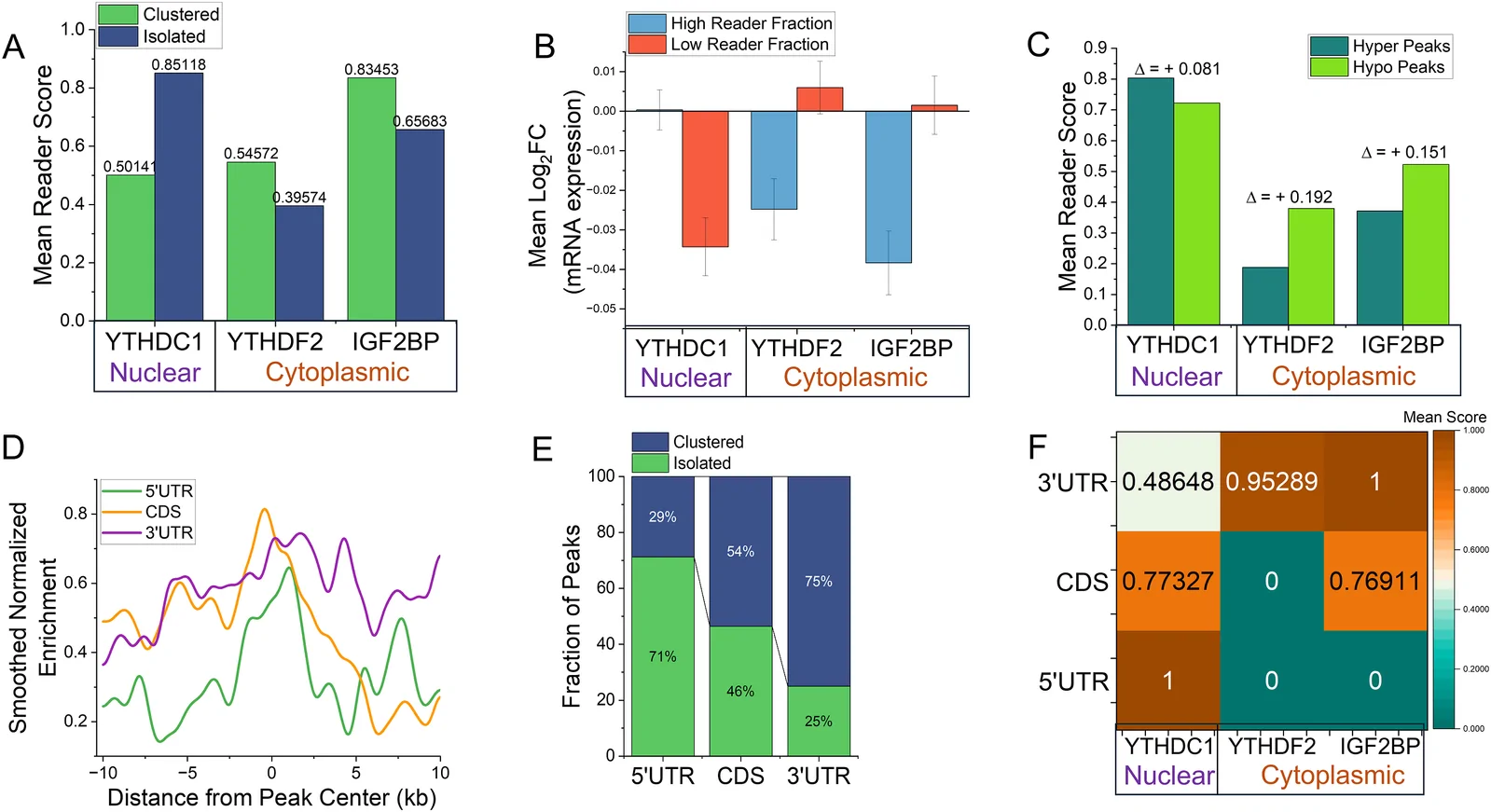
**Peak clustering encodes a reader-dependent regulatory logic.** (**A**) Fraction of peaks with reader compatibility scores in spatially clustered vs. isolated peaks. Cytoplasmic readers (YTHDF2, IGF2BP) are enriched in clusters while nuclear YTHDC1 is depleted. (**B**) Mean expression change (log2FC) for genes stratified by high vs. low reader fraction. For each reader, genes carrying at least one compatible m^6^A peak (engagement fraction > 0) were split into high- and low-engagement groups, defined as the top and bottom terciles of that reader's per-gene engagement fraction, with the middle tercile excluded; bars show the mean RNA log2 fold-change of each group, with error bars representing SEM. YTHDC1-high genes show no net mean expression change; IGF2BP-high genes are downregulated. (**C**) Reader exposure scores at hypermethylated vs. hypomethylated peaks after chronic NO treatment. YTHDC1 is enriched at hyper peaks while YTHDF2 and IGF2BP are reduced. (**D**) Average m^6^A signal topology in ±10 kb windows around peak centers, stratified by transcript region. 5′UTR peaks show sharp, isolated profiles; 3′UTR peaks show broad, clustered envelopes. (**E**) Fraction of peaks classified as isolated (>2 kb to nearest same-gene neighbor) vs. clustered, by transcript region. (**F**) Sequence-based reader prediction scores by region, showing YTHDC1 dominance at 5′UTR (100%) transitioning to YTHDF2 (95.3%) and IGF2BP (100%) dominance at 3′UTR.

Strikingly, predicted reader-compatibility patterns differed fundamentally between clustered and isolated peaks (Fig. 3A). Throughout, reader assignments are predictions based on sequence compatibility with published binding motifs, not direct measurements of reader binding under our conditions (see Methods). The cytoplasmic readers YTHDF2 (OR = 2.56) and IGF2BP (OR = 2.81) were strongly enriched within clusters, consistent with their roles in mRNA decay and stabilization, respectively, whereas nuclear YTHDC1 was depleted from clusters (OR = 0.32, all *P* ≈ 0), consistent with its function in nuclear splicing and export [52]. This spatial segregation suggests a model in which the same chemical modification could produce different functional outcomes depending on its spatial context: clustered m^6^A is predicted to be compatible with cytoplasmic decay and stability machinery, while isolated marks are predicted to favor nuclear processing functions.

Sequence-predicted reader identity tracked functional outcome (reader assignments are sequence-motif predictions, not measured occupancy). To ask whether the identity of the compatible reader, and not merely the presence of m^6^A, shapes the transcriptional consequence of modification, we assigned each gene a predicted fraction of its m^6^A marks compatible with each reader's binding motif. For each reader, we restricted the analysis to genes carrying at least one compatible m^6^A peak (engagement fraction > 0) and split these genes into a high group and a low group, defined as the top and bottom terciles of that reader's per-gene engagement fraction; the middle tercile was excluded. The bars in Fig. 3B report the mean expression change (log2FC) of each group. The contrast between groups, rather than either value alone, is the informative comparison, and it differed systematically across the three readers. Genes with a high YTHDC1 fraction showed no net mean shift in expression (n = 5281; mean log2FC = +0.0003), whereas the low-YTHDC1 group was modestly downregulated (n = 3373; mean log2FC = −0.026). Because a near-zero group mean can reflect cancellation of up- and down-regulated genes rather than a uniform absence of response, we treat this flat high-group mean as a descriptive observation, and we note only that it is compatible with YTHDC1's predominantly nuclear functions in splicing and export rather than in transcript stability.

By contrast, high-YTHDF2 genes were downregulated, whereas the low group showed a slight positive mean change (high n = 2,953, mean log2FC = −0.025; low n = 2,187, mean log2FC = +0.006), a cleaner high-versus-low separation that is consistent with the cytoplasmic decay function of YTHDF2. The IGF2BP split inverted: high-IGF2BP genes were the most downregulated of any group (n = 2814; mean log2FC = −0.038), while the low-IGF2BP group was essentially unchanged (n = 2703; mean log2FC = +0.002; Fig. 3B). This pattern runs opposite to IGF2BP's canonical stabilizing role. This suggests that because IGF2BP-compatible marks fall predominantly in the 3′UTR and chronic NO depletes 3′UTR m^6^A, the loss of these marks would remove a stabilizing influence and facilitate decay. This, however, would require direct measurement of reader occupancy. Collectively, this reader-dependent asymmetry indicates that the identity of the compatible reader, not the presence of m^6^A alone, may shape the transcriptional consequence of modification.

Chronic NO shifted the reader balance at differentially methylated peaks. At hypermethylated peaks, YTHDC1 scores were elevated relative to hypomethylated peaks (Δ = +0.081), while YTHDF2 (Δ = −0.192) and IGF2BP (Δ = −0.151) scores were reduced (Fig. 3C). This directional shift was highly significant (YTHDC1: Wilcoxon P = 3.6 × 10−27; YTHDF2: P = 7.1 × 10−91), and sequence-based motif analysis validated the pattern, with the YTHDF2-associated GGACU motif enriched 1.47-fold in YTHDF2-assigned peaks (P = 2.3 × 10−41; Supplementary Figure S1E).

Genes that change methylation without changing expression reflect insufficient architectural dose, not an active mechanism. Among genes that sustained a substantial NO-induced m^6^A change, a large subset showed no corresponding change in expression (the m^6^A-changed, expression-stable genes; classification thresholds in Methods), and we tested directly whether these genes form a mechanistically distinct population rather than the low-responding tail of the hypermethylated set. Restricting the comparison to hypermethylated genes (n = 1123; 850 unresponsive versus 273 that changed expression), we asked whether any measured feature of the mark separates the two groups. No feature did; a classifier trained on m^6^A architecture, reader compatibility, peak clustering, and transcript-region localization separated the two at a cross-validated AUC of approximately 0.55, against 0.49 for a shuffled null, indicating essentially no distinguishing signal. This non-response is not an artifact of detection power, because the unresponsive genes were, if anything more highly expressed than the responders (median baseMean 698 versus 467; Mann-Whitney P = 0.032) and were therefore measured at least as well. The only distinction was a small and diffuse one: the responding genes carried modestly more reader-compatible, spatially clustered, 3′UTR-localized m^6^A, every difference small in magnitude. We therefore interpret the lack of response not as an active mechanism of gene suppression, but simply as too little m^6^A architecture to produce an effect; these genes form a low-architecture tail in which the mark is present but insufficient to alter steady-state expression. This reinforces the central thesis that the position and architecture of m^6^A, rather than its magnitude or mere presence, determine the transcriptional outcome.

To investigate the structural basis of this reader segregation, we examined m^6^A signal topology across transcript regions. Signal profiles within ±10 kb windows around peak centers, stratified by region, revealed that 5′UTR peaks exhibit sharp, isolated enrichment signatures, a single narrow peak with rapid decay to background, whereas 3′UTR peaks display broader signal envelopes consistent with clustered modification, in which neighboring peaks contribute to a sustained enrichment plateau extending several kilobases from the center (Fig. 3D); CDS peaks showed an intermediate topology. This regional gradient in peak architecture directly parallels the reader segregation observed in Fig. 3A: isolated 5′UTR peaks match YTHDC1's monomeric binding mode, while clustered 3′UTR peaks provide the multivalent substrate required by YTHDF2 and IGF2BP.

Quantitative isolation analysis using a 2 kb threshold confirmed this striking regional gradient (Fig. 3E). At the 5′UTR, 71.2% of peaks (1331/1870) were isolated, their nearest same-gene neighbor lying more than 2 kb away, whereas only 25.0% of 3′UTR peaks (3130/12,502) were isolated, and the remaining 75.0% resided in clustered configurations; CDS peaks were evenly distributed (46.4% isolated, 53.6% clustered). Local peak density further quantified this gradient, the mean number of neighboring peaks within ±5 kb rising from 0.46 at the 5′UTR to 1.98 at the CDS and 2.54 at the 3′UTR, a 5.5-fold density increase along the 5′→3′ axis. We propose that this progressive increase in local m^6^A density corresponds to a shift in predicted reader competence: the sparse 5′UTR landscape is sequence-compatible with YTHDC1's monomeric binding mode, while the dense 3′UTR landscape would provide the clustered substrate expected for multivalent engagement by YTHDF2 and IGF2BP family members.

Sequence-based reader prediction scores confirmed this topological segregation at the molecular level (Fig. 3F). YTHDC1 was the dominant predicted reader at the 5′UTR (100% of peaks) and maintained high representation at the CDS (77.3%) but declined to 48.6% at the 3′UTR. Conversely, YTHDF2 prediction scores were negligible at the 5′UTR (0%) and CDS (0%) yet reached 95.3% at the 3′UTR, while IGF2BP scores followed a similar pattern (0% at the 5′UTR, 76.9% at the CDS, 100% at the 3′UTR). This near-complete segregation of predicted reader competence across transcript regions, together with the topological analysis showing corresponding differences in peak isolation and clustering, supports a model in which transcript position could determine reader compatibility and thus functional outcome. We focus on the 3′UTR throughout because it is the only region where m^6^A dose and concentration track downregulation (concentration vs |log2FC| ρ = +0.059, P = 4.3×10−8), consistent with reader-mediated destabilization; 5′UTR and CDS dose showed no directional association (P = 0.10 and P = 0.38).

### 3.5 There are two parallel regulatory programs: transcriptional and epitranscriptomic

**Fig. 4:**
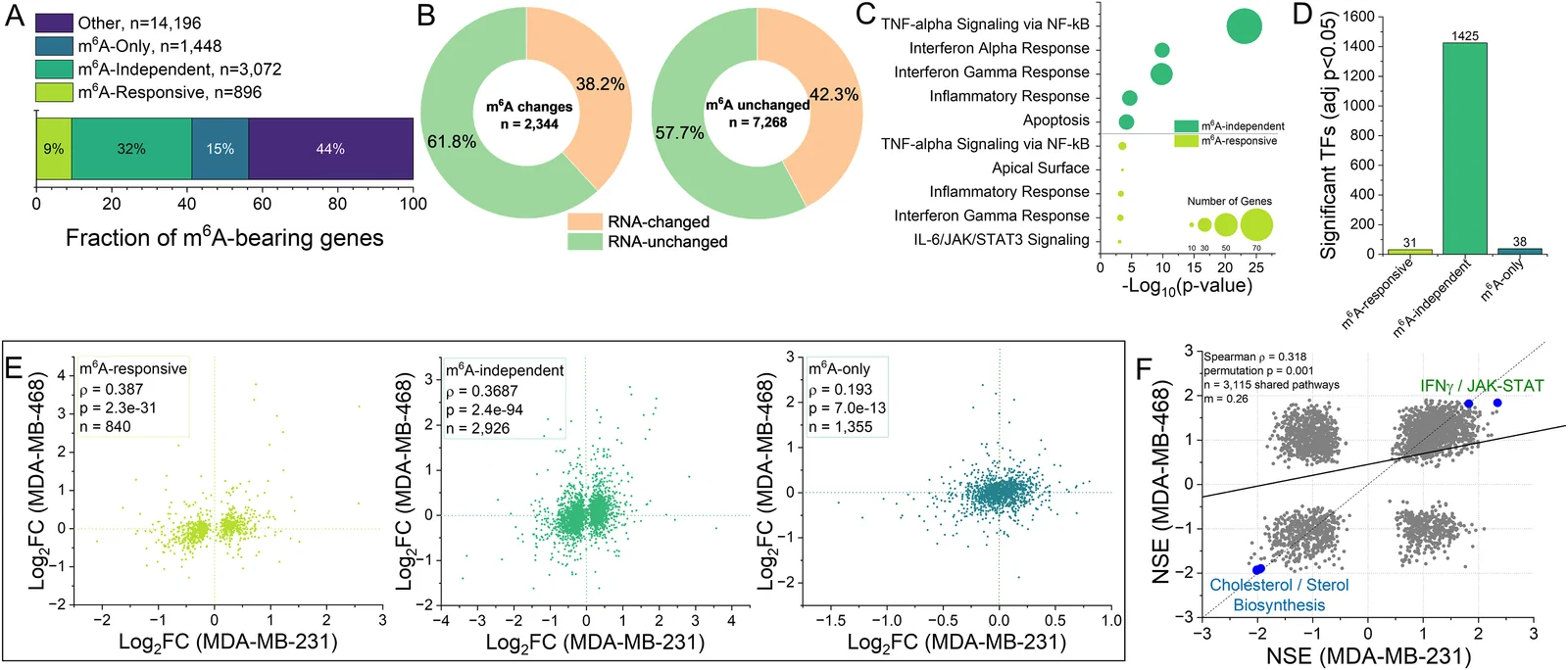
**Two parallel regulatory programs.** (**A**) Gene set sizes for the mutually exclusive programs, with "Other" denoting the unchanged-majority genes that were neither m^6^A-changed nor differentially expressed: m^6^A-responsive (896), m^6^A-independent (3,072), and m^6^A-only (1,448), shown as proportional stacked bar with gene counts and key metrics annotated. (**B**) Gene-level decoupling test: RNA-change rate among m^6^A-changed genes (n = 2344; 38.2%) versus m^6^A-unchanged genes (n = 7268; 42.3%), with the odds ratio (0.85, 95% CI [0.77–0.93]) annotated, shown alongside a companion bar for directional concordance (52.9%, not significant versus the 50% random expectation). (**C**) Pathway enrichment dot plot comparing the top MSigDB Hallmark terms between m^6^A-independent and m^6^A-responsive programs. Dot size reflects gene count; position indicates −log [10](*P*-value) (the m^6^A-independent program shows 117 significant pathway terms versus 6 for the m^6^A-responsive program). (**D**) Transcription factor enrichment showing, per program, the number of transcription factors whose curated target-gene sets are significantly enriched among that program's differentially expressed genes (adj. P < 0.05; 1425 vs. 31 vs. 38). (**E**) Cross-cell-line validation: Spearman correlation of log^2^FC values between MDA-MB-231 and MDA-MB-468 for each program. Both m^6^A-responsive (ρ = 0.387) and m^6^A-independent (ρ = 0.368) programs replicate across cell lines, while m^6^A-only genes show weaker correlation (ρ = 0.193). (**F**) Cross-line pathway concordance: per-pathway GSEA NES, MDA-MB-231 vs MDA-MB-468 (ρ = 0.32, permutation *P* = 0.001); shared-significant pathways (FDR < 0.05 both lines) highlighted.

If the m^6^A spatial code operates selectively, what drives the expression changes in genes that carry no m^6^A modification? We defined three mutually exclusive gene sets: m^6^A-responsive (896 genes; significant m^6^A change and expression change), m^6^A-independent (3072 genes; expression change without significant m^6^A change), and m^6^A-only (1448 genes; m^6^A change without expression change). The m^6^A-independent program was 3.4-fold larger than the m^6^A-responsive program, and m^6^A-responsive genes carried the highest expression magnitude (mean |log2FC| = 0.44) while m^6^A-only genes showed minimal expression change (|log2FC| = 0.15), confirming that they bear epitranscriptomic marks without transcriptional consequence. The remaining genes, labeled "Other" in Fig. 4A, are the unchanged majority that were neither significantly m^6^A-changed nor differentially expressed (Fig. 4A; Supplementary Table S1).

To test whether m^6^A change and expression change are coupled at the gene level, as a methylome-to-transcriptome relay would predict, we asked whether genes with a significant m^6^A change were enriched for expression change relative to unmodified genes. They were not: m^6^A-changed genes were no more likely to change expression than m^6^A-unchanged genes, demonstrating that the two programs are decoupled. Across 9612 genes measured in both layers, 38.2% of m^6^A-changed genes (the full 2344-gene m^6^A-changed margin, i.e. m^6^A-responsive plus m^6^A-only genes, not the 896-gene m^6^A-responsive set) also changed expression, versus 42.3% of m^6^A-unchanged genes (Fisher's exact OR = 0.85, 95% CI [0.77–0.93], *P* = 5.6 × 10^−4^), a co-occurrence at, or marginally below, the background rate rather than above it. Among genes changing in both layers, the direction of change was uncorrelated (52.9% concordant, binomial P = 0.088; Spearman r = −0.01, not significant), and this decoupling held whether a gene was counted as changed under a loose threshold, a strict threshold, or one requiring a minimum effect size. The epitranscriptomic and transcriptional responses to chronic NO are therefore statistically decoupled: m^6^A redistribution neither predicts, nor is predicted by, steady-state expression change (Fig. 4B).

Pathway enrichment analysis revealed a stark asymmetry between the two programs (Fig. 4C). The m^6^A-independent program was dominated by canonical NO signaling cascades: TNF-α signaling via NF-κB (OR = 5.68, *P* = 4.2 × 10^−22^), interferon-α response (OR = 5.41, *P* = 2.7 × 10^−9^), interferon-γ response (OR = 3.62, *P* = 2.7 × 10^−9^), and inflammatory response, 117 significant pathway terms in total (Supplementary Table S2). The m^6^A-responsive program, by contrast, showed weaker, more diffuse enrichment, IL-6/JAK/STAT3 signaling (OR = 4.57), inflammatory response (OR = 3.22), and TNF-α/NF-κB (OR = 2.85), just 6 significant pathway terms in total, consistent with post-transcriptional rather than transcription-factor-driven regulation. Because pathway enrichment is computed separately for each gene set, a pathway such as TNF-α/NF-κB can recover members in both programs; the inflammatory and NF-κB signature is nonetheless predominantly a feature of the m^6^A-independent (transcriptional) program, where it is far more strongly enriched, rather than a signature shared equally by both.

Transcription factor enrichment analysis quantified the dichotomy directly, testing how strongly each program's genes concentrate among the targets of specific transcription factors (Fig. 4D). For each program, we tested whether its differentially expressed genes were over-represented among the curated downstream target genes of individual transcription factors (ENCODE/ChEA, TRRUST, and ENCODE TF ChIP-seq libraries; see Methods); these libraries catalog the documented targets of each factor, not the genes that encode it, so a positive result means a program's changed genes are disproportionately the known targets of that factor. Binding was not measure here; what we detect is the fingerprint of established transcription-factor-to-target relationships written into the expression changes, and from that overlap we infer that the corresponding factors were active and drove the program. The presumed mechanism is binding; the evidence is the target-gene pattern in the RNA. On this basis, the target-gene sets of 1425 transcription factors were significantly enriched among the m^6^A-independent program (adj. P < 0.05), including NF-κB, HIF-1α, p53, STAT3, and SREBF1/2, whereas only 31 were enriched among the m^6^A-responsive program and 38 among the m^6^A-only program, a roughly 46-fold difference that quantifies the regulatory architecture separating transcriptional from post-transcriptional control (Supplementary Table S3).

Cross-cell-line validation demonstrated that both regulatory programs represent robust biology rather than cell-line artifacts (Fig. 4E). Expression changes in m^6^A-responsive genes correlated significantly between MDA-MB-231 and MDA-MB-468 cells (ρ = 0.387, *P* = 2.3 × 10^−31^), as did m^6^A-independent genes (ρ = 0.368, *P* = 2.4 × 10^−94^). By contrast, m^6^A-only genes showed weaker, though still highly significant, cross-line correlation (ρ = 0.193, *P* = 7.0 × 10^−13^), lower than the responsive and independent programs but non-trivial. This pattern is consistent with a partly cell-line-specific epitranscriptomic component rather than a fully conserved transcriptional program. The comparable replication strength of the m^6^A-responsive and m^6^A-independent programs (ρ ≈ 0.37–0.39) indicates that both programs represent conserved cellular responses to chronic NO, while the lower but significant correlation in m^6^A-only genes is consistent with their designation as largely silent epitranscriptomic marks whose functional consequences are not manifested at the steady-state mRNA level.

To test whether the NO-driven transcriptional program is reproducible beyond a single line, we ran pre-ranked GSEA on the MDA-MB-231 and MDA-MB-468 differential-expression rankings separately and compared per-pathway enrichment scores. The two lines were concordant (cross-line NES Spearman ρ = 0.318 over 3115 shared pathways; gene-label permutation *P* = 0.001, null mean ρ = 0.150), and seven pathways reached FDR < 0.05 in both lines, all seven agreeing in direction. The replicated program comprised two coherent axes: interferon-γ/JAK-STAT signaling was upregulated, and cholesterol/sterol-biosynthesis (SREBP-regulated) was downregulated, in both lines. TNF-α/NF-κB and angiogenesis signatures were directionally concordant across lines but reached significance only in MDA-MB-231, indicating that they are strongest in that line rather than uniformly replicated. The NO-responsive transcriptional program, anchored on interferon/JAK-STAT activation and cholesterol-biosynthesis suppression, thus shows modest but significant concordance across two independent triple-negative breast cancer lines (cross-line NES ρ = 0.318; 7 shared-significant pathways), indicating that the program is reproducible in direction rather than fully replicated (Fig. 4F; Supplementary Table S4).

### 3.6 m^6^A and miRNA compete at the 3′UTR

The 3′UTR emerged repeatedly as the primary site of m^6^A functional activity. We found that enriched peaks are demethylated under chronic NO, show the strongest concentration-dependent downregulation, and harbors the most functionally consequential co-methylation module. Because the 3′UTR is also the primary site of miRNA-mediated regulation, we hypothesized that m^6^A and miRNA binding might compete for overlapping regulatory territory, consistent with the m^6^A–miRNA axis identified in early mapping studies [56].

**Fig. 5:**
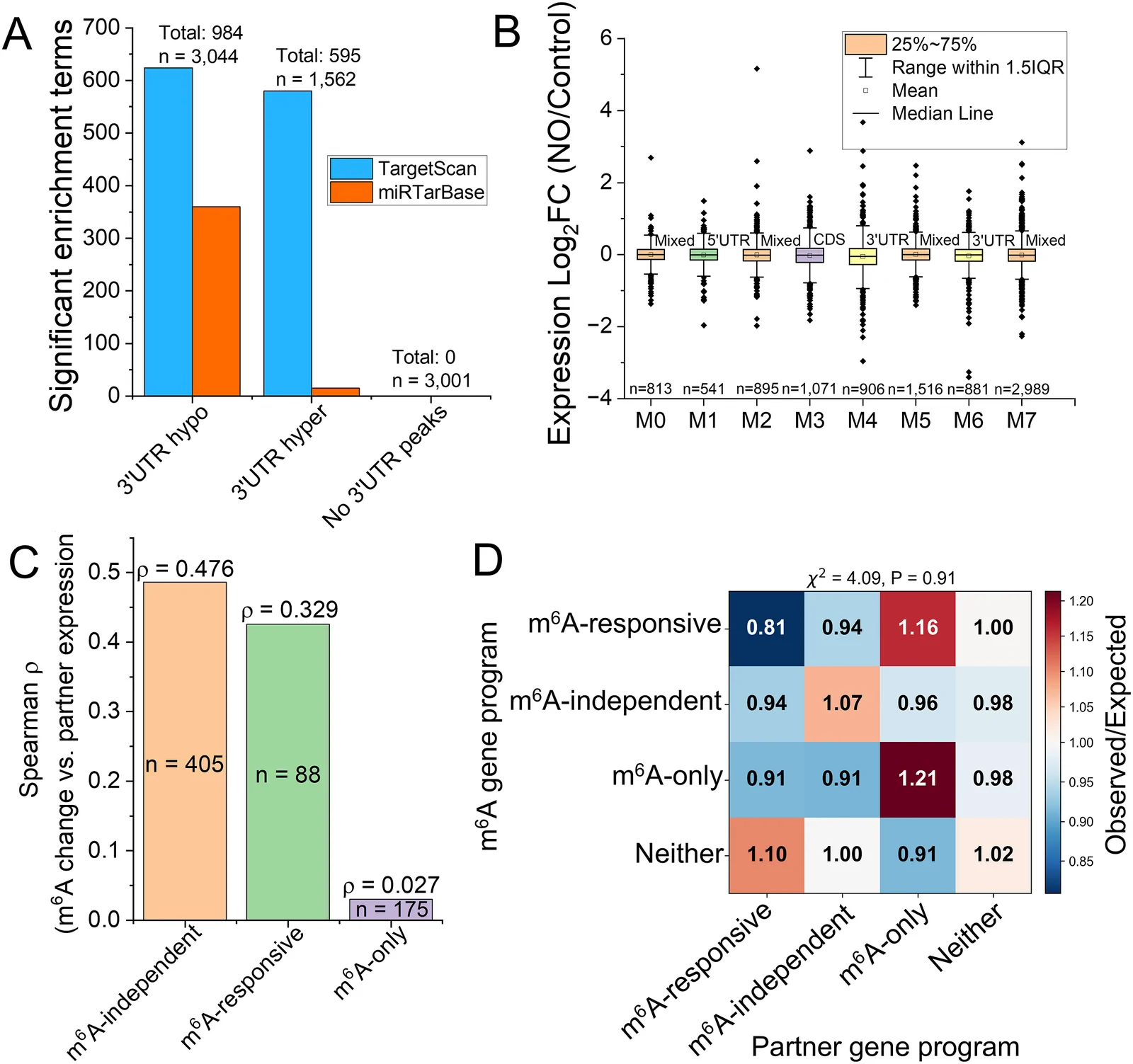
**m^6^A–miRNA competition and sense–antisense coordination at the 3′UTR.** (**A**) miRNA target-site enrichment for genes by 3′UTR m^6^A direction. 3′UTR hypomethylated genes are enriched for experimentally validated miRNA interactions (miRTarBase, 360 vs. 15 significant terms for hypermethylated genes), whereas sequence-predicted target counts are comparable (TargetScan, 624 vs. 580); genes lacking 3′UTR m^6^A peaks show no enrichment. This is a computational association based on validated interaction catalogs and is suggestive rather than demonstrated. (**B**) Expression (log_2_FC, NO vs. control) across the eight co-methylation modules (M0–M7); Module 4 (pure 3′UTR, hypomethylated, HHI = 0.996) shows the strongest downregulation of any module (median log_2_FC = −0.047). Box plots: median, 25–75% range, mean. (**C**) Sense–antisense expression coordination (Spearman ρ between m^6^A-gene and antisense-partner log2FC) stratified by the two-programs category of the m^6^A gene. Coordination is strongest for m^6^A-independent genes (ρ = 0.476, n = 405) and m^6^A-responsive genes (ρ = 0.329, n = 88), both highly significant, indicating that antisense coupling tracks the transcriptional program. The m^6^A-only group (n = 175) is defined by minimal expression change and carries too little expression variance to support a coordination test, so its near-zero correlation (ρ = 0.027, P = 0.55) cannot be assessed rather than absent. (**D**) Heatmap of antisense pair counts, with each pair classified by the regulatory program of one gene (rows) and of its antisense partner (columns); cells give the number of pairs. The two programs are independent (χ^2^ = 4.09, P = 0.91), so antisense pairing does not link the transcriptional and epitranscriptomic programs.

Consistent with this model, we applied a purely computational test of miRNA target-site enrichment that drew predicted targets from TargetScan and experimentally validated targets from miRTarBase, not an experimental miRNA assay. Genes with 3′UTR hypomethylation were strongly enriched for experimentally validated miRNA interactions relative to genes with 3′UTR hypermethylation (360 versus 15 significant miRTarBase terms), whereas the density of sequence-predicted target sites was comparable between the two groups (624 versus 580 significant TargetScan terms; Fig. 5A). The comparable predicted-site counts are expected, because loss of m^6^A exposes pre-existing seed-match sites rather than creating new sequence motifs, so a sequence scan should recover similar site densities in both groups. The validated-interaction catalog, however, is a static compilation assembled from prior studies under unrelated conditions, and its coverage tracks how extensively each gene has been studied; because the hypomethylated genes are the more-studied downregulated set, this enrichment is consistent with, but does not on its own demonstrate NO-induced exposure of miRNA sites, and it remains a purely computational association rather than direct evidence of altered miRNA occupancy. Genes lacking 3′UTR m^6^A peaks showed zero significant miRNA enrichment, indicating that the association is specific to the 3′UTR m^6^A landscape rather than a genome-wide phenomenon (Fig. 5A)**.**

### 3.7 Most m^6^A localization modules are expression-neutral; the pure-3′UTR hypomethylated module is the exception

Having established that 3′UTR hypomethylation tracks transcript downregulation, we asked whether this coupling is a general property of m^6^A methylation or is specific to a particular m^6^A architecture. To address this, we partitioned all genes by their m^6^A architectural features, namely transcript-region localization, spatial concentration, and methylation direction, and tested each resulting module against expression as an independent outcome (Methods). This partition resolved eight co-methylation modules defined by dominant m^6^A localization, comprising modules dominated by 5′UTR, CDS, intronic, 3′UTR, or dispersed multi-region methylation, with each module's architecture detailed in Supplementary Table S5. Of these eight modules, only one showed a significant change in expression: Module 4 (906 genes), defined by pure 3′UTR localization (mean 3′UTR fraction = 0.998), near-perfect spatial concentration (HHI = 0.996), and hypomethylation, was downregulated relative to all other modules (median log2FC = −0.047, Wilcoxon P = 1 × 10−4; Fig. 5B); the across-module difference (Kruskal–Wallis P = 4.2 × 10−3) was driven almost entirely by this module. The remaining modules, including those organized around 5′UTR, CDS, and intronic methylation, were expression-neutral. This specificity argues that the link between m^6^A and transcript downregulation is a property of the pure-3′UTR, hypomethylated architecture rather than of methylation generally, and that the coupling does not emerge from a biased subset but survives partitioning of the entire gene set. Together, the downregulation of the 3′UTR-hypomethylated module is consistent with a competition model in which m^6^A occupancy at the 3′UTR blocks miRNA access, such that loss of 3′UTR m^6^A following chronic NO exposure could expose these miRNA sites and facilitate miRNA-mediated repression; we note that this model is suggested by, not demonstrated by, the present computational data (Fig. 5A and B).

### 3.8 Sense–antisense coordination tracks the transcriptional program and does not bridge the two programs

Many genes are transcribed together with an overlapping opposite-strand gene; we refer to this overlapping opposite-strand gene as the antisense "partner," and we quantify whether a gene and its partner change expression together, stratified by the regulatory program of the m^6^A-bearing gene (Fig. 5C). Natural antisense transcripts (NATs) can regulate their sense partners through transcriptional interference, chromatin remodeling, and dsRNA-mediated mechanisms [65,66]. Given that NO activates two parallel regulatory programs, we asked whether sense–antisense pairs might bridge the epitranscriptomic and transcriptional programs, providing a mechanism for crosstalk. Parsing the Ensembl v109 annotation for overlapping opposite-strand gene pairs identified 2697 antisense pairs with a median genomic overlap of 2449 bp (Supplementary Table S6). Of these, 987 pairs (36.6%) had both genes bearing m^6^A peaks, 1270 (47.1%) were asymmetric (one m^6^A-marked, one not), and 440 (16.3%) had neither gene marked. Notable antisense pairs, miRNA host-gene analysis, and summary statistics are provided in Supplementary Table S6.

Antisense expression coordination was highly significant among genes that change expression, and it tracked the regulatory program of the m^6^A-bearing gene (Fig. 5C). The m^6^A-independent genes showed the strongest coordination with their antisense partners, with a Spearman ρ = 0.476 among m^6^A-independent pairs specifically (P = 2.6 × 10−24; concordance rate 66.7%), followed by m^6^A-responsive genes (ρ = 0.329, P = 1.7 × 10−3; concordance 67.0%). The m^6^A-only genes, defined by minimal expression change, carry too little expression variation to support a coordination test; their log2FC variance is 2.5- to 3.7-fold smaller than that of the other categories and barely overlaps it, so their near-zero correlation (ρ = 0.027, P = 0.55) reflects range restriction rather than absent coupling and cannot be read as evidence that their antisense partners are uncoordinated. Among the categories that can be assessed, the difference in concordance rates was highly significant (χ2 = 19.3, P = 2.4 × 10−4), and this hierarchy was robust across all 15 threshold combinations tested in sensitivity analysis.

We next asked whether antisense partners belong to connected regulatory programs. Each gene falls into one of two programs: transcriptional (its expression changes with NO but its m^6^A does not) or epitranscriptomic (its m^6^A is NO-responsive). If antisense pairing linked these two layers, an epitranscriptomic gene would preferentially pair with a transcriptional partner, forming a cross-program "bridge." It does not. Across antisense pairs, the two partners' programs were independent (χ^2^ = 4.09, P = 0.91), and bridge pairs occurred no more often than chance predicts (37 observed versus 39.4 expected; Fig. 5D). Antisense pairing therefore does not connect the transcriptional and epitranscriptomic programs.

This category dependence is biologically coherent. The m^6^A-independent genes are driven by shared transcription-factor programs (NF-κB, TNF-α), and the antisense partners of these genes likely respond to the same transcriptional activators, accounting for their coordinated expression. The m^6^A-only genes, by contrast, carry epitranscriptomic marks without an expression consequence, so their expression barely changes by definition and their antisense coordination cannot be assessed; their near-flat log2FC leaves too little variance for a meaningful correlation, and the absence of a measurable signal in this group therefore reflects an untestable comparison rather than demonstrated decoupling. The m^6^A-only genes were nonetheless modestly enriched for antisense partners relative to other categories (OR = 1.15, *P* = 0.028), suggesting that antisense pairing may contribute to expression buffering in this group [67]. Collectively, these results show that the antisense regulatory layer reinforces the transcriptional program, while the contingency analysis of program membership across antisense pairs reveals no detectable bridge to the epitranscriptomic program (Fig. 5D), consistent with these two parallel regulatory systems being decoupled rather than fully coupled.

## 4 Discussion

Our comprehensive reanalysis of m^6^A-RIP-seq and RNA-seq data from breast cancer cells subjected to chronic NO exposure (10 days) reveals that the epitranscriptomic response is not a simple increase in global methylation, but a spatially organized regulatory code in which modification location, concentration, clustering, and multiplicity encode distinct functional outcomes. This reframes our original finding, that NO inhibits FTO to increase m^6^A [10], from a scalar model in which increasing methylation yields more effect to an architectural model in which the spatial pattern determines the outcome. This distinction matters because conventional m^6^A biology treats the modification as topologically stable, concentrated near the stop codon and within the 3′UTR, and the perturbations studied to date overwhelmingly change the amount of m^6^A rather than the positions it occupies. Chronic NO is unusual in that it reorganizes where m^6^A resides, decreasing the 3′UTR while increasing the mark across the 5′UTR and coding sequence, so that the epitranscriptome is spatially reprogrammed rather than simply amplified. These findings are among the first to define such a spatial reprogramming of the mRNA m^6^A landscape, and they establish chronic NO as a driver of a genuinely unconventional mode of epitranscriptomic regulation that operates outside the scalar logic of demethylase inhibition; this paradigm reframes NO not as an signaling molecule that merely raises m^6^A abundance, but as one that rewrites its positional code.

### 4.1 Physiological rationale for the chronic NO model

To date most *in vitro* NO-exposure cell culture studies have been conducted within the time frame of hours (1-24h) which is useful to model cell phenotype and gene expression changes. However, these model systems may not accurately capture sustained responses to NO that are exhibited within tumors where parenchymal cells are exposed to chronic NO exposure (days). The concentration and duration used here were chosen to model sustained, physiologically relevant NO exposure rather than an acute bolus. We previously demonstrated that NO inhibits recombinant FTO across a steady-state range of approximately 500 nM to 30 μM [NO]ss by coordinating its catalytic non-heme iron, and that in cells DETA/NO delivering steady-state NO of only 10–500 nM is sufficient to drive a concentration-dependent increase in m^6^A that persists across the 10-day chronic paradigm [10]. The 100 μM donor concentration, replenished every 48 h, therefore places cells at the low end of the FTO-inhibitory NO range and within the sustained, low-micromolar output characteristic of inducible NOS2 in inflamed and tumor tissue rather than the high-picomolar bursts of canonical signaling [14,68]. This exposure is not restricted to bulk methylation: we have shown that the same 10-day chronic NO paradigm reorganizes the DNA methylome through inhibition of the TET/ALKBH2 demethylases [69], indicating that the chosen regimen is a reproducible window for Fe(II)/2-oxoglutarate demethylase inhibition rather than an arbitrary dose. The model is further grounded in endogenous NO, since NOS2-expressing MDA-MB-231 raise m^6^A through self-generated NO in a manner reversed by NOS inhibition [10,70], and high tumoral NOS2 in turn predicts poor survival in estrogen-receptor-negative and triple-negative breast cancer [25,71], defining the NO-producing tumor context that this model is intended to represent. Direct intracellular NO across the 10-day course was not quantified, and a precise intratumoral NO concentration for human breast tumors is not available for numerical comparison; sustained exposure is inferred from the replenishment schedule against the donor half-life, and physiological relevance is argued from the delivered steady-state range and the endogenous-NO experiments rather than from a measured tumor concentration.

Signaling effects of NO are a function of both the concentration and the duration of NO exposure [4]. The amount of NO used in these studies was to model lower-concentrations at longer durations which is most representative of sustained NOS2 output in inflamed and tumor tissue, and it plausibly approaches the reorganization ceiling set by the finite pool of available methylation sites. We have shown that NO inhibits the Fe(II)/2-oxoglutarate demethylases only above a minimum steady-state concentration [10,69], consistent with the concentration-dependent mechanism of NO signaling in which discrete thresholds concentrations engage distinct molecular targets [1]; below this threshold m^6^A is unchanged irrespective of how long NO is present, whereas above it the inhibition is reversible and the mark accumulates over time as writer activity outpaces the blocked erasers. The extent of remodeling should therefore scale with the integrated supra-threshold exposure, the product of concentration and duration above the threshold, rather than with peak concentration alone, so that a lower concentration sustained across days and a higher concentration experienced briefly can converge on a comparable steady-state m^6^A landscape once each exceeds the inhibitory threshold.

### 4.2 The spatial code concept

The analogy to the histone code is instructive and is grounded in our own recent work. We recently demonstrated that chronic NO generates a coordinated histone post-translational modification signature consistent with chromatin compaction, driven by NO-mediated inhibition of the same Fe(II)/2-oxoglutarate-dependent demethylase family that includes FTO and ALKBH5, and linked to tumor-permissive gene silencing [72]. Together with the present epitranscriptomic findings, this places the m^6^A spatial code within a broader program of NO-driven epigenetic reprogramming spanning histones and mRNA. Just as specific combinations of histone modifications at different positions recruit distinct effector complexes, we find that m^6^A marks at different transcript positions recruit different reader proteins and produce different expression outcomes. The 5′UTR + CDS signature is associated with upregulation, consistent with cap-independent translation [73] and translation-elongation effects, whereas the 3′UTR-only signature drives downregulation, consistent with YTHDF2-mediated decay [61] and miRNA competition. The CDS gradient, in which the distal coding region behaves functionally like the 3′UTR, has not been previously described and suggests that the CDS–3′UTR boundary is a functional transition zone rather than a sharp demarcation.

### 4.3 Origin of 3′UTR depletion

How eraser inhibition produces a localized loss of m^6^A at the 3′UTR rather than a uniform gain remains to be established. One possibility is that the apparent 3′UTR depletion reflects an enlarged pool of newly synthesized (nascent) transcripts that have not yet been methylated; under such a scenario, elevated transcription could present as relative 3′UTR hypomethylation even as bulk methylation rises elsewhere. This would require metabolic-labeling or nascent-RNA approaches to test directly.

### 4.4 Reader context determines the type of effect, not whether a gene responds

Every reader assignment in this study is a sequence-based prediction of motif compatibility rather than a measurement of reader occupancy under NO, so the reader-dependent outcomes we describe are mechanistic hypotheses to be tested by direct occupancy mapping rather than demonstrated binding. The underlying principle is nonetheless well established and bidirectional: the same m^6^A mark can be routed to decay when read by YTHDF2 or to stabilization when read by the IGF2BP family [54,62], and reader selection of this kind governs the fate of named oncogenes, including IGF2BP- and YBX1-dependent stabilization of MYC and BCL2 [74], while stimulus-driven relocation of readers can switch the outcome at 5′UTR marks during the stress response. A positional or density shift that changes which reader is locally competent is thus a mechanistically grounded route by which NO could redirect transcript fate, and our prediction that clustered 3′UTR marks are compatible with cytoplasmic YTHDF2 and IGF2BP engagement whereas sparse 5′UTR marks favor nuclear YTHDC1 is consistent with this framework; a direct demonstration that local m^6^A density or spacing, as opposed to presence or absence, toggles the reader remains to be shown.

The most mechanistically informative finding is the segregation of readers between clustered and isolated peaks. Cytoplasmic readers are predicted to preferentially associate with clustered m^6^A; this includes YTHDF2 for decay [75] and IGF2BP for stabilization. Nuclear YTHDC1, by contrast, is predicted to be compatible with isolated peaks. This division provides a structural explanation for why the same mark can produce opposite effects, stabilization or decay, depending on context.

Reader context explains the type of effect, but it does not explain why some methylated genes do not respond at all. We tested this directly in the m^6^A-changed, expression-stable class, restricting the comparison to hypermethylated genes. No measured feature of the mark separated the genes that stayed expression-stable from those that changed, and reader identity in particular did not; sequence compatibility with YTHDC1 tracked hypermethylation generally rather than the expression-stable subset specifically. We therefore interpret the non-response as the absence of a sufficient architectural dose, not as active reader-mediated protection. These genes are the low-architecture tail, in which the mark is present but not configured to be transmitted to expression; they are not transcripts shielded by a nuclear reader. The practical implication for interpreting epitranscriptomic data still holds, and is in fact sharpened. Not all m^6^A changes are equal. A change scored only by its magnitude or presence, without regard to its position and architecture, will systematically overestimate the functional effect.

### 4.5 Two parallel, decoupled programs

The central architectural finding of this study is that chronic NO activates two parallel regulatory programs that are decoupled at the gene level: a dominant transcription-factor-driven transcriptional program (1425 TFs, 117 pathways) and a smaller, spatially organized epitranscriptomic program (31 TFs, 6 pathways). The decoupling is not merely a difference in pathway enrichment: at the gene level, m^6^A change and expression change co-occur at or below the background rate (odds ratio = 0.85, 95% CI [0.77–0.93]), and among the genes that do change in both layers, the sign of the methylation change does not match the sign of the expression change (52.9% concordant, not significant), indicating that the two programs are decoupled rather than sequentially linked, and that the occurrence and magnitude of an m^6^A change do not propagate into a coupled transcriptional change. This decoupling of occurrence and magnitude coexists with, rather than contradicts, the positional association we observe in the responding population (Fig. 1B and E): among transcripts that do change, the location of the mark along the transcript is associated with the direction of the change, whereas whether a transcript is methylated, and by how much, does not predict whether or how much its expression changes.

### 4.6 A replicated functional program downstream of the spatial code

The transcriptional output of chronic NO is itself reproducible in direction across two TNBC cell lines, a modest but significant concordance. Pre-ranked GSEA performed independently in MDA-MB-231 and MDA-MB-468 was significantly concordant (NES ρ = 0.318, with 7 pathways at FDR < 0.05 in both lines; permutation *P* = 0.001). The two cleanly replicated axes, interferon-γ/JAK-STAT activation and suppression of cholesterol/sterol biosynthesis, are biologically coherent for chronic NO. Interferon/JAK-STAT engagement is consistent with the inflammatory milieu of sustained NO flux, while SREBP-dependent sterol-biosynthesis suppression links NO to lipid-metabolic reprogramming in these cells. The canonical TNF-α/NF-κB inflammatory signature, by contrast, was strongest in MDA-MB-231 and only directionally concordant in MDA-MB-468, indicating line-dependent amplitude within a shared directional response. This replicated interferon/cholesterol program represents the most defensible functional consequence of the NO-remodeled epitranscriptomic landscape that our data support. These two replicated axes carry direct implications for tumor phenotype. Sustained interferon-γ/JAK-STAT activation is consistent with the pro-inflammatory, immunomodulatory milieu that accompanies high NOS2 output in aggressive breast tumors and that has been linked to tumor-permissive signaling and poor outcome, whereas coordinate suppression of SREBP-driven cholesterol and sterol biosynthesis points to a reprogramming of membrane-lipid and mevalonate metabolism of the kind that supports proliferative and metastatic phenotypes, so we interpret the NO-remodeled landscape not as a neutral rearrangement of marks but as a program whose replicated functional output, inflammatory/immune activation together with lipid-metabolic reprogramming, is aligned with the aggressive biology of NO-producing triple-negative breast cancer. Although JAK–STAT signaling features in both programs, IL-6/JAK/STAT3 within the m^6^A-responsive set and interferon-γ-driven JAK-STAT within the transcriptional program, these reflect distinct upstream inputs converging on shared downstream machinery; the decoupling we report is at the level of individual gene responses, not mutual exclusivity of signaling pathways.

### 4.7 m^6^A–miRNA competition

The enrichment of validated miRNA interactions among 3′UTR hypomethylated genes is carried by experimentally curated target databases and is therefore a suggestive computational association rather than a demonstrated mechanism; sequence-predicted target density did not differ appreciably between the hypomethylated and hypermethylated sets. With that caveat in mind, the association is compatible with a competition model. If m^6^A occupancy blocks miRNA access, then chronic NO-induced hypomethylation at the 3′UTR could expose miRNA binding sites that the m^6^A mark normally shields, potentially linking the spatial code to miRNA-mediated repression. In this model, an m^6^A mark sitting on or near a miRNA seed-match site sterically occludes RISC loading, so that the NO-driven loss of 3′UTR m^6^A removes that protection and facilitates miRNA binding at sites that were previously masked. The result would be a feed-forward loop in which loss of m^6^A reduces reader-mediated stabilization while simultaneously enabling miRNA-mediated silencing.

A related question is whether the non-coding transcriptome, and miRNAs in particular, are themselves NO-regulated under these conditions. We did not profile small RNAs here, but NO is an established regulator of miRNA abundance: our own surveys of NO-dependent epigenetics enumerate microRNA levels alongside histone and DNA methylation as a third regulated layer [76,77], and specific NO- and NOS2-dependent miRNA changes have been documented, including miR-29b/c and cGMP-dependent induction of miR-155 [78], with a reciprocal miR-744/eNOS circuit operating in MDA-MB-231 themselves [79]. These NO–miRNA effects intersect the epitranscriptome directly, because m^6^A deposited on primary miRNAs licenses their Microprocessor cropping through DGCR8 recognition [80]; since NO raises m^6^A by inhibiting FTO and ALKBH5, NO-driven m^6^A gain on pri-miRNAs offers a plausible mechanistic bridge by which the redistribution we describe could feed into altered miRNA biogenesis. We present this as a hypothesis for future small-RNA and Ago-occupancy studies rather than a conclusion supported by the present data.

### 4.8 Sense–antisense coordination as an independent regulatory layer

A complementary observation is that genes which gain m^6^A tend to gain it on both the sense and the antisense strand of an overlapping pair, so the modification is laid down symmetrically across the duplex rather than on one strand alone. Whether this symmetric gain translates into coordinated expression, however, depends entirely on the regulatory program the gene belongs to, not on the m^6^A change itself. Our analysis of 2697 sense–antisense pairs revealed that expression coordination is real but category-dependent: m^6^A-independent genes show the strongest antisense coordination (ρ = 0.476), consistent with shared transcriptional control. Representative pairs illustrate how this coordination manifests biologically. Within the inflammatory response, IL6 and its stabilizing antisense lncRNA IL6-AS1 are coordinately induced, reinforcing the interferon/inflammatory signature; the invasion-associated protease MMP1, which gains m^6^A under NO, is coordinately induced with the overlapping WTAP pseudogene WTAPP1, a provocative link given WTAP's role in the m^6^A writer complex; and at the keratin locus KRT10, which itself sustains a genuine NO-dependent loss of m^6^A, the sense gene and its antisense partner KRT10-AS1 move concordantly. These examples, drawn from the notable-pair catalog in Supplementary Table S6, indicate that antisense partners plausibly contribute to the magnitude of the transcriptional response at individual inflammatory and invasion-associated loci. Critically, we found no evidence that antisense pairing bridges the two parallel programs, since bridge pairs were not enriched (ratio = 0.94, *P* = 0.81), consistent with mechanistic divergence between the transcriptional and epitranscriptomic programs. The m^6^A-only genes are defined by minimal expression change, so their antisense coordination cannot be assessed; their near-flat expression leaves too little variance to support a correlation, and this group is therefore uninformative for coordination rather than evidence that the epitranscriptomic program is decoupled from sense–antisense regulation.

### 4.9 Therapeutic implications

In cancer, FTO inhibitors are currently under investigation and would primarily affect the epitranscriptomic program, which represents a minority of NO-responsive genes and operates through diffuse post-transcriptional mechanisms, while the dominant transcriptional program driven by NF-κB, TNF-α, and p53 would remain largely unaffected by m^6^A-targeted intervention. In this regard, combination approaches, targeting both the transcriptional and the epitranscriptomic arm, may be necessary for comprehensive modulation of NO-driven cancer phenotypes. Contrary to a linear cascade model, m^6^A redistribution does not propagate into a coupled transcriptional change; the cascade hypothesis was tested directly and rejected.

### 4.10 Limitations

Several limitations should be considered. We additionally performed spectral-count proteomics on 10-day NO-treated cells (n = 3); these data were underpowered to resolve protein-level changes and were not analyzed further. Reader scores are sequence-based predictions, not condition-specific binding measurements, and validating actual reader occupancy changes would require eCLIP-seq in NO-treated cells. Although we have demonstrated global increases in m^6^A in over ten different cell lines exposed to NO, the limited cell lines for m^6^A analysis (MDA-MB-231) limits generalizability. Cross-cell-line validation with MDA-MB-468 RNA-seq provided partial support. Finally, all analyses derive from a single 10-day steady-state timepoint, so we cannot establish the temporal order of the two programs or infer causality directly; a change in m^6^A distribution could precede, follow, or occur independently of the transcriptional response. We note that m^6^A is deposited co-transcriptionally and is largely fixed before a transcript's cytoplasmic life^84,85^, and that it specifies downstream stability and decay through reader engagement, so a positional m^6^A change is expected on kinetic grounds to precede rather than follow the resulting steady-state abundance change, and stimulus-driven m^6^A remodeling can occur rapidly [73]; a resolved time course will nonetheless be required to test this directly. Because MeRIP-seq localizes m^6^A to peaks of finite width rather than to single nucleotides, the precise position and spacing of individual marks carry technical uncertainty, and several of our conclusions rest on peak location and clustering. The spatial-polarization enrichments that anchor the positional code, 5′UTR and CDS hypermethylation with 3′UTR hypomethylation, nonetheless retain their direction, magnitude, and significance across the full range of effect-size thresholds tested and are computed over the aggregate peak population rather than individually significant peaks (Fig. S2), indicating that the positional patterns are robust to peak-calling uncertainty rather than artifacts of a particular threshold or of individual peak boundaries. Many of the positional associations we report are individually small at the level of a single gene, accounting for less than 0.1% of per-gene variance in expression change; we interpret them accordingly, not as strong per-transcript predictors but as reproducible, directional trends that are real and interpretable at the transcriptome scale, where the position of m^6^A shapes the aggregate direction of expression change even though the magnitude of any single mark does not.

### 4.11 Future directions

The spatial code model generates several testable predictions. YTHDC1 and YTHDF2 eCLIP-seq in NO-treated versus control cells would directly test the reader segregation hypothesis, and Ago2-RIP-seq would determine whether 3′UTR hypomethylated sites gain miRNA occupancy. Ribosome profiling (Ribo-seq) could reveal translational effects invisible to standard RNA-seq, particularly for the m^6^A-changed, expression-stable genes. Testing the spatial code framework in other chronic exposure models that modulate FTO activity, such as obesity and hypoxia, would establish whether the code is specific to NO or represents a general principle of epitranscriptomic regulation. Finally, single-molecule resolution techniques such as miCLIP or direct RNA sequencing by Oxford Nanopore could validate the clustering and co-methylation modules defined here.

A further question is whether the positional code operates in other tissues exposed to chronic NO such as the vasculature, where the exposure regime would significantly differ from the one modeled here. Endothelial cells experience continuous eNOS-derived NO that is chronic in duration but lower in concentration than sustained NOS2 output, thus under the concentration–duration relationship outlined above it remains an empirical question whether these conditions may cross the threshold for demethylase inhibition. Endothelium also retains an intact sGC/cGMP/PKG arm that is dispensable for the DNIC-mediated eraser inhibition described here, suggesting the possibility of a writer-directed input alongside the eraser-directed one characterized here. Importantly, cardiovascular and cardiometabolic disease is often characterized by NO insufficiency rather than excess, such that the framework would predict a mirror image of the redistribution reported here, and m^6^A-RIP-seq across a graded chronic NO range in endothelial cells would test whether the positional code, and its decoupling from the transcriptional program, generalizes beyond the tumor context and is primed for future study.

In conclusion, our analysis reveals that chronic NO exposure does not simply increase m^6^A abundance but rather rewires the epitranscriptomic landscape into a spatially organized regulatory code. The location, concentration, clustering, and multiplicity of m^6^A marks, not their intensity, determine functional outcome through selective reader recruitment. This positional code operates alongside, and is decoupled from, canonical transcription-factor-driven gene regulation, establishing the epitranscriptome as a parallel regulatory layer with its own spatially encoded logic.

## CRediT authorship contribution statement

**Sara Shayan:** Formal analysis, Methodology, Writing – review & editing. **Hannah Petraitis Kuschman:** Data curation, Formal analysis, Writing – original draft, Writing – review & editing. **Christopher G. Kevil:** Conceptualization, Writing – review & editing. **Douglas D. Thomas:** Conceptualization, Formal analysis, Funding acquisition, Project administration, Resources, Supervision, Visualization, Writing – original draft.

## Declaration of generative AI use

Generative AI tools (Anthropic) were used during the preparation of this manuscript to assist with data visualization and statistical analysis. The authors reviewed and edited all AI-generated content and take full responsibility for the content of the publication.

## Declaration of competing interests

H.K. is currently employed by Sterne, Kessler, Goldstein & Fox PLLC, Washington, DC 20005.

## Figures and Tables

**figs1:**
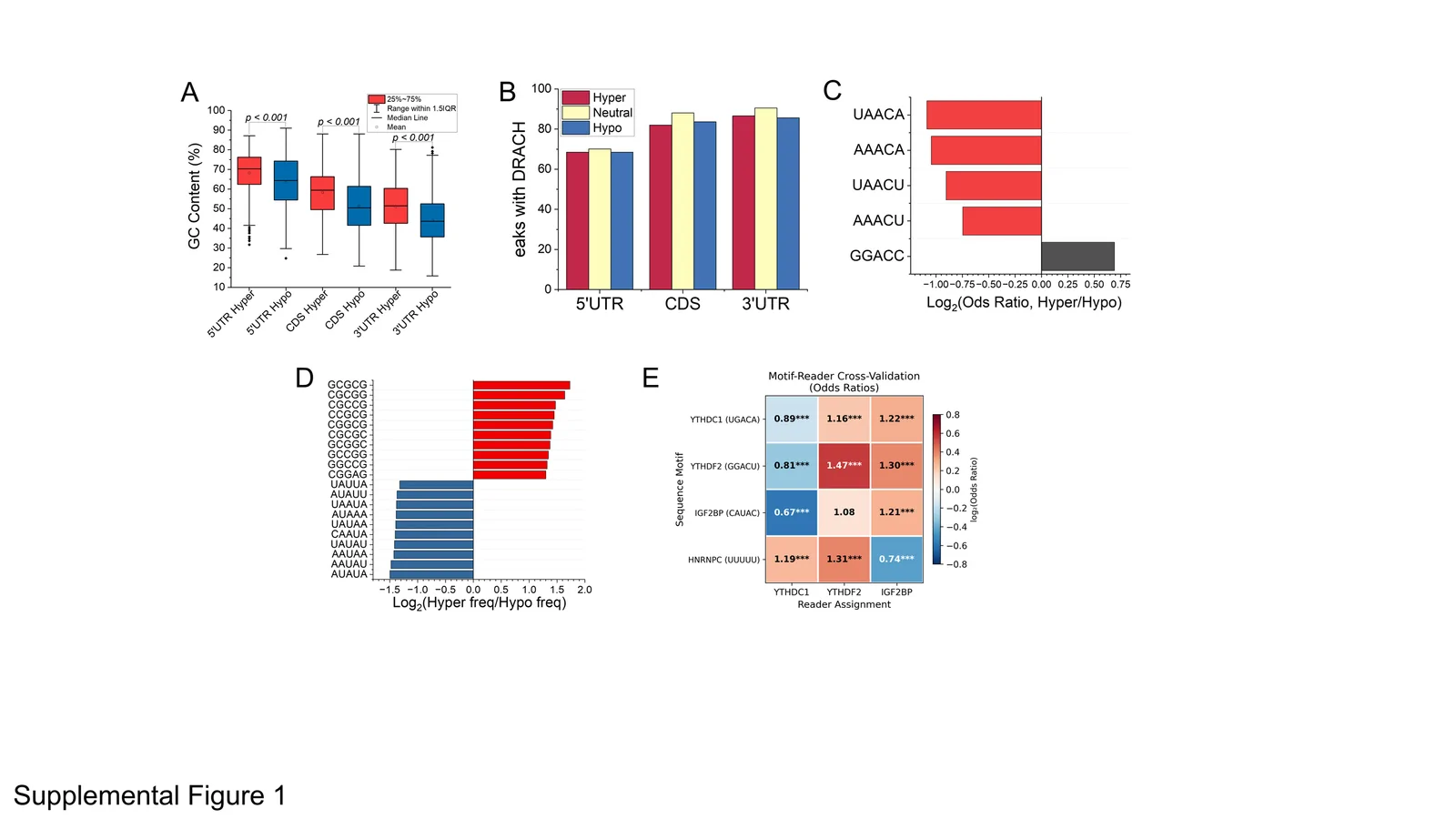


**figs2:**
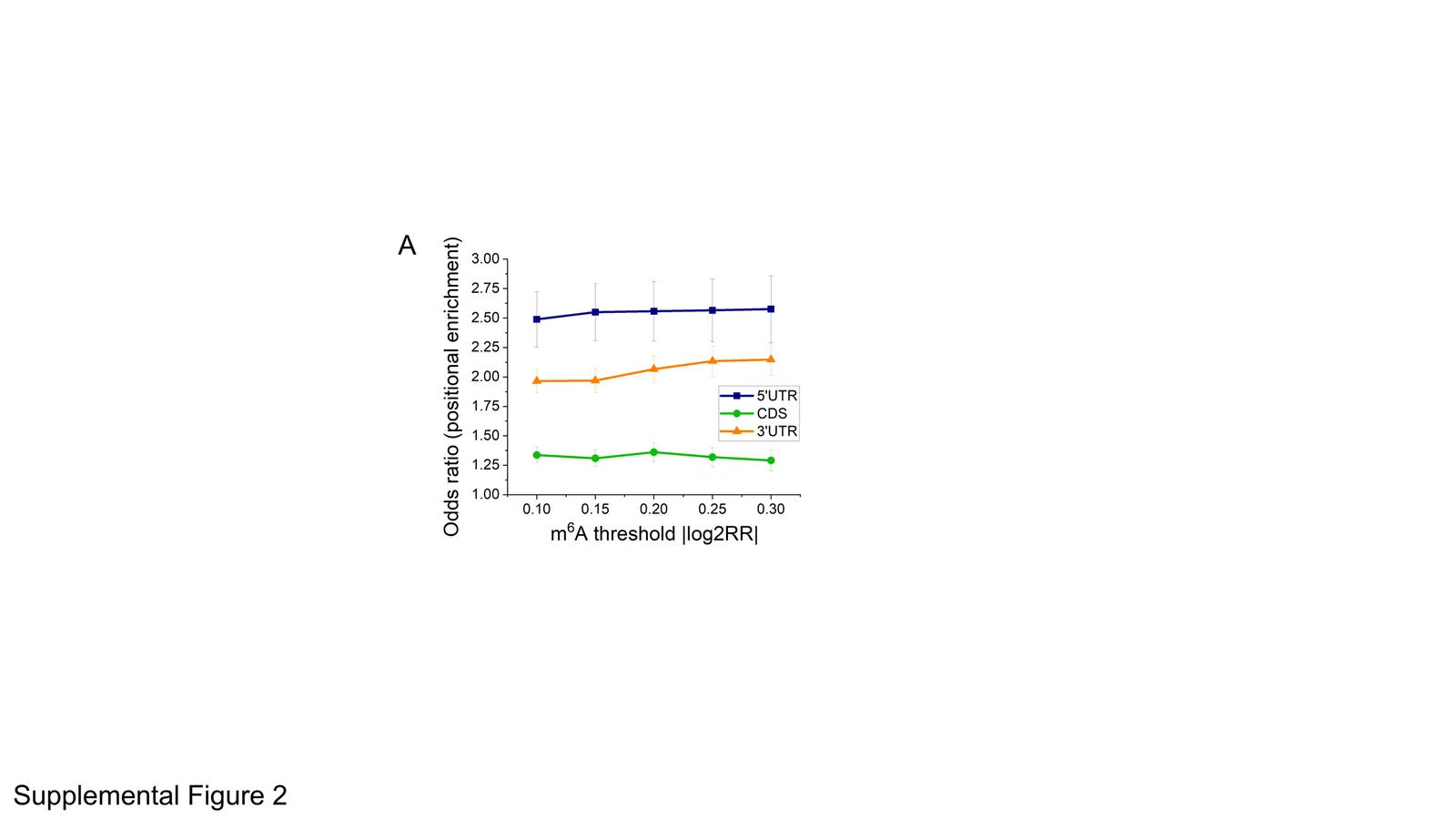


## Data Availability

RNA-sequencing and m^6^A-MeRIP-sequencing data are stored in the NCBI GEO repository under accession number GSE236272. Source data are provided with this paper.

## References

[1] Thomas D.D., Espey M.G., Ridnour L.A., Hofseth L.J., Mancardi D., Harris C.C., Wink D.A. (2004). Hypoxic inducible factor 1alpha, extracellular signal-regulated kinase, and p53 are regulated by distinct threshold concentrations of nitric oxide. Proc. Natl. Acad. Sci. U. S. A..

[2] Hickok J.R., Vasudevan D., Jablonski K., Thomas D.D. (2013). Oxygen dependence of nitric oxide-mediated signaling. Redox Biol..

[3] Somasundaram V., Basudhar D., Bharadwaj G., No J.H., Ridnour L.A., Cheng R.Y.S., Fujita M., Thomas D.D., Anderson S.K., McVicar D.W., Wink D.A. (2019). Molecular mechanisms of nitric oxide in cancer progression, signal transduction, and metabolism. Antioxid. Redox Signaling.

[4] Thomas D.D. (2015). Breathing new life into nitric oxide signaling: a brief overview of the interplay between oxygen and nitric oxide. Redox Biol..

[5] Hickok J.R., Vasudevan D., Thatcher G.R., Thomas D.D. (2012). Is S-nitrosocysteine a true surrogate for nitric oxide?. Antioxid. Redox Signaling.

[6] Stomberski C.T., Hess D.T., Stamler J.S. (2019). Protein S-Nitrosylation: determinants of specificity and enzymatic regulation of S-Nitrosothiol-Based signaling. Antioxid. Redox Signaling.

[7] Dao V.T., Elbatreek M.H., Deile M., Nedvetsky P.I., Guldner A., Ibarra-Alvarado C., Godecke A., Schmidt H. (2020). Non-canonical chemical feedback self-limits nitric oxide-cyclic GMP signaling in health and disease. Sci. Rep..

[8] Hickok J.R., Vasudevan D., Antholine W.E., Thomas D.D. (2013). Nitric oxide modifies global histone methylation by inhibiting Jumonji C domain-containing demethylases. J. Biol. Chem..

[9] Vasudevan D., Hickok J.R., Bovee R.C., Pham V., Mantell L.L., Bahroos N., Kanabar P., Cao X.J., Maienschein-Cline M., Garcia B.A., Thomas D.D. (2015). Nitric oxide regulates gene expression in cancers by controlling histone posttranslational modifications. Cancer Res..

[10] Kuschman H.P., Palczewski M.B., Hoffman B., Menhart M., Wang X., Glynn S., Islam A., Benevolenskaya E.V., Thomas D.D. (2023). Nitric oxide inhibits FTO demethylase activity to regulate N(6)-methyladenosine mRNA methylation. Redox Biol..

[11] Palczewski M.B, Kuschman H.P., Hoffman B.M., Kathiresan V., Yang H., Glynn S., Wilson D.L., Kool E.T., Montfort W.R., Chang J., Petenkaya A., Chronis C., Cundari T.R., Sappa S., Islam K., McVicar D.W., Fan Y., Chen Q., Meerzaman D., Sierk M., Thomas D.D. (2025). Nitric oxide inhibits ten-eleven translocation DNA demethylases to regulate 5mC and 5hmC across the genome. Nat. Commun..

[12] Yamashita A.M.S., Ancillotti M.T.C., Rangel L.P., Fontenele M., Figueiredo-Freitas C., Possidonio A.C., Soares C.P., Sorenson M.M., Mermelstein C., Nogueira L. (2017). Balance between S-nitrosylation and denitrosylation modulates myoblast proliferation independently of soluble guanylyl cyclase activation. Am. J. Physiol. Cell Physiol..

[13] Roundtree I.A., Evans M.E., Pan T., He C. (2017). Dynamic RNA modifications in gene expression regulation. Cell.

[14] Thomas D.D., Heinecke J.L., Ridnour L.A., Cheng R.Y., Kesarwala A.H., Switzer C.H., McVicar D.W., Roberts D.D., Glynn S., Fukuto J.M. (2015). Signaling and stress: the redox landscape in NOS2 biology. Free Radic. Biol. Med..

[15] McGinity C.L., Palmieri E.M., Somasundaram V., Bhattacharyya D.D., Ridnour L.A., Cheng R.Y.S., Ryan A.E., Glynn S.A., Thomas D.D., Miranda K.M. (2021). Nitric oxide modulates metabolic processes in the tumor immune microenvironment. Int. J. Mol. Sci..

[16] Hickok J.R., Thomas D.D. (2010). Nitric oxide and cancer therapy: the emperor has NO clothes. Curr. Pharm. Des..

[17] Vasudevan D., Thomas D.D. (2014). Insights into the diverse effects of nitric oxide on tumor biology. Vitam. Horm..

[18] Loibl S., Buck A., Strank C., von Minckwitz G., Roller M., Sinn H.P., Schini-Kerth V., Solbach C., Strebhardt K., Kaufmann M. (2005). The role of early expression of inducible nitric oxide synthase in human breast cancer. Eur. J. Cancer.

[19] De Paepe B., Verstraeten V.M., De Potter C.R., Bullock G.R. (2002). Increased angiotensin II type-2 receptor density in hyperplasia, DCIS and invasive carcinoma of the breast is paralleled with increased iNOS expression. Histochem. Cell Biol..

[20] Heinecke J.L., Ridnour L.A., Cheng R.Y., Switzer C.H., Lizardo M.M., Khanna C., Glynn S.A., Hussain S.P., Young H.A., Ambs S., Wink D.A. (2014). Tumor microenvironment-based feed-forward regulation of NOS2 in breast cancer progression. Proc. Natl. Acad. Sci. U. S. A..

[21] Switzer C.H., Cheng R.Y., Ridnour L.A., Glynn S.A., Ambs S., Wink D.A. (2012). Ets-1 is a transcriptional mediator of oncogenic nitric oxide signaling in estrogen receptor-negative breast cancer. Breast Cancer Res..

[22] Ridnour L.A., Barasch K.M., Windhausen A.N., Dorsey T.H., Lizardo M.M., Yfantis H.G., Lee D.H., Switzer C.H., Cheng R.Y., Heinecke J.L. (2012). Nitric oxide synthase and breast cancer: role of TIMP-1 in NO-mediated Akt activation. PLoS One.

[23] Switzer C.H., Ridnour L.A., Cheng R., Heinecke J., Burke A., Glynn S., Ambs S., Wink D.A. (2012). S-Nitrosation mediates multiple pathways that lead to tumor progression in Estrogen receptor-negative breast cancer. Forum Immunopathol. Dis. Ther..

[24] Switzer C.H., Glynn S.A., Ridnour L.A., Cheng R.Y., Vitek M.P., Ambs S., Wink D.A. (2011). Nitric oxide and protein phosphatase 2A provide novel therapeutic opportunities in ER-negative breast cancer. Trends Pharmacol. Sci..

[25] Glynn S.A., Boersma B.J., Dorsey T.H., Yi M., Yfantis H.G., Ridnour L.A., Martin D.N., Switzer C.H., Hudson R.S., Wink D.A. (2010). Increased NOS2 predicts poor survival in estrogen receptor-negative breast cancer patients. J. Clin. Investig..

[26] Prueitt R.L., Boersma B.J., Howe T.M., Goodman J.E., Thomas D.D., Ying L., Pfiester C.M., Yfantis H.G., Cottrell J.R., Lee D.H. (2007). Inflammation and IGF-I activate the Akt pathway in breast cancer. International journal of cancer. Journal international du cancer.

[27] Cheng R.Y.S., Ridnour L.A., Wink A.L., Gonzalez A.L., Femino E.L., Rittscher H., Somasundaram V., Heinz W.F., Coutinho L., Rangel M.C. (2023). Interferon-gamma is quintessential for NOS2 and COX2 expression in ER(-) breast tumors that lead to poor outcome. Cell Death Dis..

[28] Somasundaram V., Ridnour L.A., Cheng R.Y., Walke A.J., Kedei N., Bhattacharyya D.D., Wink A.L., Edmondson E.F., Butcher D., Warner A.C. (2022). Systemic Nos2 depletion and cox inhibition limits TNBC disease progression and alters lymphoid cell spatial orientation and density. Redox Biol..

[29] Liu P.F., Zhao D.H., Qi Y., Wang J.G., Zhao M., Xiao K., Xie L.X. (2018). The clinical value of exhaled nitric oxide in patients with lung cancer. Clin. Res. J.

[30] Zhang L., Liu J., Wang X., Li Z., Zhang X., Cao P., She X., Dai Q., Tang J., Liu Z. (2014). Upregulation of cytoskeleton protein and extracellular matrix protein induced by stromal-derived nitric oxide promotes lung cancer invasion and metastasis. Curr. Mol. Med..

[31] Gao X., Xuan Y., Benner A., Anusruti A., Brenner H., Schottker B. (2019). Nitric oxide metabolites and lung cancer incidence: a matched case-control study nested in the ESTHER cohort. Oxid. Med. Cell. Longev..

[32] Lee K.M., Kang D., Park S.K., Berndt S.I., Reding D., Chatterjee N., Chanock S., Huang W.Y., Hayes R.B. (2009). Nitric oxide synthase gene polymorphisms and prostate cancer risk. Carcinogenesis.

[33] Erlandsson A., Carlsson J., Andersson S.O., Vyas C., Wikstrom P., Andren O., Davidsson S., Rider J.R. (2018). High inducible nitric oxide synthase in prostate tumor epithelium is associated with lethal prostate cancer. Scand J Urol.

[34] Burke A.J., McAuliffe J.D., Natoni A., Ridge S., Sullivan F.J., Glynn S.A. (2022). Chronic nitric oxide exposure induces prostate cell carcinogenesis, involving genetic instability and a pro-tumorigenic secretory phenotype. Nitric Oxide.

[35] Fahey J.M., Korytowski W., Girotti A.W. (2019). Upstream signaling events leading to elevated production of pro-survival nitric oxide in photodynamically-challenged glioblastoma cells. Free Radic. Biol. Med..

[36] Altinoz M.A., Elmaci I. (2018). Targeting nitric oxide and NMDA receptor-associated pathways in treatment of high grade glial tumors. Hypotheses for nitro-memantine and nitrones. Nitric Oxide.

[37] Puglisi M.A., Cenciarelli C., Tesori V., Cappellari M., Martini M., Di Francesco A.M., Giorda E., Carsetti R., Ricci-Vitiani L., Gasbarrini A. (2015). High nitric oxide production, secondary to inducible nitric oxide synthase expression, is essential for regulation of the tumour-initiating properties of colon cancer stem cells. J. Pathol..

[38] de Oliveira G.A., Cheng R.Y.S., Ridnour L.A., Basudhar D., Somasundaram V., McVicar D.W., Monteiro H.P., Wink D.A. (2017). Inducible nitric oxide synthase in the carcinogenesis of gastrointestinal cancers. Antioxidants Redox Signal..

[39] Drehmer D., Mesquita Luiz J.P., Hernandez C.A.S., Alves-Filho J.C., Hussell T., Townsend P.A., Moncada S. (2022). Nitric oxide favours tumour-promoting inflammation through mitochondria-dependent and -independent actions on macrophages. Redox Biol..

[40] Goncalves D.A., Xisto R., Goncalves J.D., da Silva D.B., Moura Soares J.P., Icimoto M.Y., Sant'Anna C., Gimenez M., de Angelis K., Llesuy S. (2019). Imbalance between nitric oxide and superoxide anion induced by uncoupled nitric oxide synthase contributes to human melanoma development. Int. J. Biochem. Cell Biol..

[41] Massi D., De Nisi M.C., Franchi A., Mourmouras V., Baroni G., Panelos J., Santucci M., Miracco C. (2009). Inducible nitric oxide synthase expression in melanoma: implications in lymphangiogenesis. Mod. Pathol..

[42] Lopez-Rivera E., Jayaraman P., Parikh F., Davies M.A., Ekmekcioglu S., Izadmehr S., Milton D.R., Chipuk J.E., Grimm E.A., Estrada Y. (2014). Inducible nitric oxide synthase drives mTOR pathway activation and proliferation of human melanoma by reversible nitrosylation of TSC2. Cancer Res..

[43] Eller-Borges R., Rodrigues E.G., Teodoro A.C.S., Moraes M.S., Arruda D.C., Paschoalin T., Curcio M.F., da Costa P.E., Do Nascimento I.R., Calixto L.A. (2023). Bradykinin promotes murine melanoma cell migration and invasion through endogenous production of superoxide and nitric oxide. Nitric Oxide.

[44] Wang R., Li Y., Tsung A., Huang H., Du Q., Yang M., Deng M., Xiong S., Wang X., Zhang L. (2018). iNOS promotes CD24(+)CD133(+) liver cancer stem cell phenotype through a TACE/ADAM17-dependent notch signaling pathway. Proc. Natl. Acad. Sci. U. S. A..

[45] Park Y.H., Shin H.J., Kim S.U., Kim J.M., Kim J.H., Bang D.H., Chang K.T., Kim B.Y., Yu D.Y. (2013). iNOS promotes HBx-induced hepatocellular carcinoma via upregulation of JNK activation. Biochem. Biophys. Res. Commun..

[46] Wiener D., Schwartz S. (2021). The epitranscriptome beyond m(6)A. Nat. Rev. Genet..

[47] Sendinc E., Shi Y. (2023). RNA m6A methylation across the transcriptome. Mol. Cell.

[48] Meyer K.D., Saletore Y., Zumbo P., Elemento O., Mason C.E., Jaffrey S.R. (2012). Comprehensive analysis of mRNA methylation reveals enrichment in 3' UTRs and near stop codons. Cell.

[49] Liu S., Zhuo L., Wang J., Zhang Q., Li Q., Li G., Yan L., Jin T., Pan T., Sui X. (2020). METTL3 plays multiple functions in biological processes. Am. J. Cancer Res..

[50] Relier S., Rivals E., David A. (2022). The multifaceted functions of the Fat mass and obesity-associated protein (FTO) in normal and cancer cells. RNA Biol..

[51] Schmidl D., Jonasson N.S.W., Menke A., Schneider S., Daumann L. (2022). Spectroscopic and in vitro investigations of Fe2+/alpha-Ketoglutarate-dependent enzymes involved in nucleic acid repair and modification. Chembiochem.

[52] Xiao W., Adhikari S., Dahal U., Chen Y.S., Hao Y.J., Sun B.F., Sun H.Y., Li A., Ping X.L., Lai W.Y. (2016). Nuclear m(6)A reader YTHDC1 regulates mRNA splicing. Mol. Cell.

[53] Chen L., Gao Y., Xu S., Yuan J., Wang M., Li T., Gong J. (2023). N6-methyladenosine reader YTHDF family in biological processes: structures, roles, and mechanisms. Front. Immunol..

[54] Huang H., Weng H., Sun W., Qin X., Shi H., Wu H., Zhao B.S., Mesquita A., Liu C., Yuan C.L. (2018). Recognition of RNA N(6)-methyladenosine by IGF2BP proteins enhances mRNA stability and translation. Nat. Cell Biol..

[55] Zaccara S., Jaffrey S.R. (2020). A unified model for the function of YTHDF proteins in regulating m(6)A-Modified mRNA. Cell.

[56] Alarcon C.R., Goodarzi H., Lee H., Liu X., Tavazoie S., Tavazoie S.F. (2015). HNRNPA2B1 is a mediator of m(6)A-Dependent nuclear RNA processing events. Cell.

[57] Baksh S.C., Finley L.W.S. (2021). Metabolic coordination of cell fate by alpha-ketoglutarate-dependent dioxygenases. Trends Cell Biol..

[58] Cui X., Meng J., Zhang S., Chen Y., Huang Y. (2016). A novel algorithm for calling mRNA m6A peaks by modeling biological variances in MeRIP-seq data. Bioinformatics.

[59] Muzellec B., Telenczuk M., Cabeli V., Andreux M. (2023). PyDESeq2: a python package for bulk RNA-seq differential expression analysis. Bioinformatics.

[60] Love M.I., Huber W., Anders S. (2014). Moderated estimation of fold change and dispersion for RNA-seq data with DESeq2. Genome Biol..

[61] Wang X., Lu Z., Gomez A., Hon G.C., Yue Y., Han D., Fu Y., Parisien M., Dai Q., Jia G. (2014). N6-methyladenosine-dependent regulation of messenger RNA stability. Nature.

[62] Wang X., Zhao B.S., Roundtree I.A., Lu Z., Han D., Ma H., Weng X., Chen K., Shi H., He C. (2015). N(6)-methyladenosine modulates messenger RNA translation efficiency. Cell.

[63] Subramanian A., Tamayo P., Mootha V.K., Mukherjee S., Ebert B.L., Gillette M.A., Paulovich A., Pomeroy S.L., Golub T.R., Lander E.S., Mesirov J.P. (2005). Gene set enrichment analysis: a knowledge-based approach for interpreting genome-wide expression profiles. Proc. Natl. Acad. Sci. U. S. A..

[64] Fang Z., Liu X., Peltz G. (2023). GSEApy: a comprehensive package for performing gene set enrichment analysis in Python. Bioinformatics.

[65] Faghihi M.A., Wahlestedt C. (2009). Regulatory roles of natural antisense transcripts. Nat. Rev. Mol. Cell Biol..

[66] Pelechano V., Steinmetz L.M. (2013). Gene regulation by antisense transcription. Nat. Rev. Genet..

[67] Wight M., Werner A. (2013). The functions of natural antisense transcripts. Essays Biochem..

[68] Miranda K.M., Ridnour L.A., Cheng R.Y.S., Wink D.A., Thomas D.D. (2023). The chemical biology of NO that regulates oncogenic signaling and metabolism: NOS2 and its role in inflammatory disease. Crit. Rev. Oncog..

[69] Palczewski M.B., Kuschman H.P., Hoffman B.M., Kathiresan V., Yang H., Glynn S.A., Wilson D.L., Kool E.T., Montfort W.R., Chang J. (2025). Nitric oxide inhibits ten-eleven translocation DNA demethylases to regulate 5mC and 5hmC across the genome. Nat. Commun..

[70] Palczewski M.B., Kuschman H.P., Bovee R., Hickok J.R., Thomas D.D. (2021). Vorinostat exhibits anticancer effects in triple-negative breast cancer cells by preventing nitric oxide-driven histone deacetylation. Biol. Chem..

[71] Granados-Principal S., Liu Y., Guevara M.L., Blanco E., Choi D.S., Qian W., Patel T., Rodriguez A.A., Cusimano J., Weiss H.L. (2015). Inhibition of iNOS as a novel effective targeted therapy against triple-negative breast cancer. Breast Cancer Res..

[72] Shayan S., Vasudevan D., Thomas D.D. (2026). Nitric oxide generates a coordinated histone modification signature consistent with chromatin compaction, linked to methyl-modifying enzyme dysregulation and tumor-permissive gene silencing. Redox Biol..

[73] Zhou J., Wan J., Gao X., Zhang X., Jaffrey S.R., Qian S.B. (2015). Dynamic m(6)A mRNA methylation directs translational control of heat shock response. Nature.

[74] Feng M., Xie X., Han G., Zhang T., Li Y., Li Y., Yin R., Wang Q., Zhang T., Wang P. (2021). YBX1 is required for maintaining myeloid leukemia cell survival by regulating BCL2 stability in an m6A-dependent manner. Blood.

[75] Du H., Zhao Y., He J., Zhang Y., Xi H., Liu M., Ma J., Wu L. (2016). YTHDF2 destabilizes m(6)A-containing RNA through direct recruitment of the CCR4-NOT deadenylase complex. Nat. Commun..

[76] Socco S., Bovee R.C., Palczewski M.B., Hickok J.R., Thomas D.D. (2017). Epigenetics: the third pillar of nitric oxide signaling. Pharmacol. Res..

[77] Mathe E., Nguyen G.H., Funamizu N., He P., Moake M., Croce C.M., Hussain S.P. (2012). Inflammation regulates microRNA expression in cooperation with p53 and nitric oxide. Int. J. Cancer.

[78] Yuhas Y., Berent E., Ashkenazi S. (2014). Effect of nitric oxide on microRNA-155 expression in human hepatic epithelial cells. Inflamm. Res..

[79] El Kilany F.H., Youness R.A., Assal R.A., Gad M.Z. (2021). miR-744/eNOS/NO axis: a novel target to halt triple negative breast cancer progression. Breast Dis..

[80] Alarcon C.R., Lee H., Goodarzi H., Halberg N., Tavazoie S.F. (2015). N6-methyladenosine marks primary microRNAs for processing. Nature.

